# Machine Learning for Prediction of Stable Warfarin Dose in US Latinos and Latin Americans

**DOI:** 10.3389/fphar.2021.749786

**Published:** 2021-10-29

**Authors:** Heidi E. Steiner, Jason B. Giles, Hayley Knight Patterson, Jianglin Feng, Nihal El Rouby, Karla Claudio, Leiliane Rodrigues Marcatto, Leticia Camargo Tavares, Jubby Marcela Galvez, Carlos-Alberto Calderon-Ospina, Xiaoxiao Sun, Mara H. Hutz, Stuart A. Scott, Larisa H. Cavallari, Dora Janeth Fonseca-Mendoza, Jorge Duconge, Mariana Rodrigues Botton, Paulo Caleb Junior Lima Santos, Jason H. Karnes

**Affiliations:** ^1^ Department of Pharmacy Practice and Science, University of Arizona College of Pharmacy, Tucson, AZ, United States; ^2^ Department of Pharmacotherapy and Translational Research and Center for Pharmacogenomics and Precision Medicine, University of Florida College of Pharmacy, Gainesville, FL, United States; ^3^ Department of Pharmaceutical Sciences, University of Puerto Rico School of Pharmacy, Medical Sciences Campus, San Juan, PR, United States; ^4^ Instituto do Coracao do Hospital das Clinicas da Faculdade de Medicina, HCFMUSP, University of São Paulo, São Paulo, Brazil; ^5^ Faculty of Science, School of Biological Sciences, Monash University, Melbourne, VIC, Australia; ^6^ Center for Research in Genetics and Genomics–CIGGUR, GENIUROS Research Group, School of Medicine and Health Sciences, Universidad Del Rosario, Bogotá, Colombia; ^7^ Department of Epidemiology Biostatistics, University of Arizona College of Public Health, Tucson, AZ, United States; ^8^ Departament of Genetics, Universidade Federal do Rio Grande do Sul, Porto Alegre, Brazil; ^9^ Department of Pathology, Stanford University, Clinical Genomics Laboratory, Stanford Health Care, Palo Alto, CA, United States; ^10^ Cells, Tissues and Genes Laboratory, Hospital de Clínicas de Porto Alegre, Porto Alegre, Brazil; ^11^ Department of Pharmacology, Escola Paulista de Medicina, Universidade Federal de São Paulo, EPM-Unifesp, São Paulo, Brazil; ^12^ Department of Biomedical Informatics, Vanderbilt University Medical Center, Nashville, TN, United States

**Keywords:** pharmacogenetics, machine learning, anticoagulant, warfarin, Latino, Hispanic

## Abstract

Populations used to create warfarin dose prediction algorithms largely lacked participants reporting Hispanic or Latino ethnicity. While previous research suggests nonlinear modeling improves warfarin dose prediction, this research has mainly focused on populations with primarily European ancestry. We compare the accuracy of stable warfarin dose prediction using linear and nonlinear machine learning models in a large cohort enriched for US Latinos and Latin Americans (ULLA). Each model was tested using the same variables as published by the International Warfarin Pharmacogenetics Consortium (IWPC) and using an expanded set of variables including ethnicity and warfarin indication. We utilized a multiple linear regression model and three nonlinear regression models: Bayesian Additive Regression Trees, Multivariate Adaptive Regression Splines, and Support Vector Regression. We compared each model’s ability to predict stable warfarin dose within 20% of actual stable dose, confirming trained models in a 30% testing dataset with 100 rounds of resampling. In all patients (*n* = 7,030), inclusion of additional predictor variables led to a small but significant improvement in prediction of dose relative to the IWPC algorithm (47.8 versus 46.7% in IWPC, *p* = 1.43 × 10^−15^). Nonlinear models using IWPC variables did not significantly improve prediction of dose over the linear IWPC algorithm. In ULLA patients alone (*n* = 1,734), IWPC performed similarly to all other linear and nonlinear pharmacogenetic algorithms. Our results reinforce the validity of IWPC in a large, ethnically diverse population and suggest that additional variables that capture warfarin dose variability may improve warfarin dose prediction algorithms.

## Introduction

Despite the availability of direct oral anticoagulants (DOACs), warfarin remains a commonly prescribed drug in the United States and Latin America. Although a highly effective anticoagulant, warfarin’s small therapeutic window and high inter-patient dose variability make it a leading cause of adverse drug events. While warfarin use may decline due to the requirement for regular clinical monitoring, a significant proportion of the population is likely to continue warfarin use preferentially over use of DOACs. Clinical concerns with DOACs continue to limit their use, including fewer indications than warfarin, concerns about bleeding risk and renal function, availability and cost of reversal agents, and contraindication in valvular heart disease (Nielsen et al., 2015; Verdecchia et al., 2016; Mendoza-Sanchez et al., 2018; Vinogradova et al., 2018; Zhu et al., 2018). This is especially true for medically underserved patients, including US Latino and Latin American (ULLA) patients, who may have access barriers to newer agents because of high costs and copays(Kirley et al., 2012; Shahin and Giacomini, 2020). Given the long track record of warfarin use in clinical practice, its affordable cost, and limited clinical utility of DOACs in special populations, warfarin is likely to continue to be preferentially used over DOACs in a substantial proportion of the population(Shahin et al., 2011; Barnes et al., 2015; Arwood et al., 2017).

In order to reduce warfarin-associated adverse drug events, warfarin stable dose prediction algorithms have been developed that incorporate clinical and genetic factors(Gage et al., 2008; Botton et al., 2011; Grossi et al., 2014; Alzubiedi and Saleh, 2016). Variables used in dose prediction algorithms account for approximately 50% of the variability in warfarin dose. However, these models, such as the International Warfarin Pharmacogenetics Consortium model (IWPC), were derived from populations with largely white participants and very small ULLA populations, including less than 1% ULLA in the IWPC cohort (International Warfarin Pharmacogenetics Consortium et al., 2009). Thus, it is possible that variability in warfarin stable dose requirements in ULLA patients may not be accurately modelled in commonly-used dose prediction algorithms, making these models potentially less effective for these patients(Kimmel et al., 2013; French et al., 2016; Johnson et al., 2017). This is particularly concerning since medically underserved patients, including disproportionately high ULLA patients, are at high risk for poor outcomes during warfarin treatment (White et al., 2006; Shen et al., 2007; Writing Group Members et al., 2016). Warfarin dosing in ULLA populations, which can have a mosaic-like ancestry that is admixed with the genomes of European, African, and Native American ancestors, (Wang et al., 2008) may be improved by developing algorithms trained with data from ULLA patients (Kaye et al., 2017). Recommended warfarin stable dose algorithms are based on multiple linear regression models(Johnson et al., 2017). Given that the relationship between warfarin dose and predictor variables is complex, nonlinear modeling strategies have been tested in warfarin dose prediction(Grossi et al., 2014; Liu et al., 2015; Roche-Lima et al., 2020). Non-parametric machine learning models are potentially powerful alternatives to linear parametric models in that they lack many of the assumptions of linear regression and they are flexible enough to fit virtually any curve in the data. However, the term machine learning technically applies to all models used in this analysis. The main aims of this study were to determine the validity of IWPC in ULLA patients and to apply machine learning to assess the accuracy of warfarin dose prediction with the published IWPC algorithm, a novel linear model, and three types of nonlinear models.

## Methods

### Study Populations

We analyzed publicly available data from IWPC combined with multiple cohorts of ULLA patients treated with stable doses of warfarin, creating a large ethnically diverse population. First, we obtained IWPC open access data from The Pharmacogenomics Knowledgebase (PharmGKB) website (https://www.pharmgkb.org/downloads, accessed December 2020), which contains data on 5,700 warfarin users recruited through 22 collaborative research groups from four continents (International Warfarin Pharmacogenetics Consortium et al., 2009). The IWPC cohort has been previously described in detail (International Warfarin Pharmacogenetics Consortium et al., 2009). The dataset contains detailed de-identified, curated data on demographics, clinical features, and genotypes for single nucleotide polymorphisms (SNPs) in *CYP2C9* and *VKORC1*.

In this study, all ULLA patients self-reported Hispanic or Latino ethnicity or were recruited in a Latin American country. Herein, Hispanic ethnicity is used to refer to an individual with Spanish-speaking culture or origin and Latino ethnicity is used to refer to an individual with culture or origin from a Latin American country. Hispanic and Latino ethnicities are not mutually exclusive and are self-reported regardless of race. We chose the term ULLA to be inclusive of study participants who are not currently residing in Latin America (i.e., may not identify as Latin American), who are not Spanish speaking (i.e., do not identify as Hispanic), and who do not follow U.S. social constructs (i.e., may not identify as ethnically Hispanic/Latino). In addition to patients self-reporting as ULLA, patients also self-reported Black or White race. Given the incredible diversity within ULLA patients, statistical methods described below also included evaluation of the influence of self-reported race, as well as country of enrollment, within the ULLA cohort.

The cohort of ULLA patients comprised 1,757 warfarin-treated patients with Hispanic or Latino ethnicity recruited through research groups in North and South America. Each of the cohorts have been previously described (Perini et al., 2008; Lubitz et al., 2010; Botton et al., 2011; Bress et al., 2012; Santos et al., 2015; Duconge et al., 2016; Galvez et al., 2018; El Rouby et al., 2020). Data for a total of 411 self-reported Latinos were collected in North America, consisting of participant data from the University of Arizona (*n* = 76), University of Illinois at Chicago (*n* = 54), University of Puerto Rico (*n* = 260), and Icahn School of Medicine at Mount Sinai (*n* = 21). The South American cohorts were enrolled in Brazil from the University of São Paulo (*n* = 663) and Federal University of Rio Grande do Sul (*n* = 533) and in Colombia from the Hospital Universitario Mayor in Bogotá (*n* = 150). All participants were recruited while taking a stable dose of warfarin, defined as taking a consistent warfarin dose for two or more visits and achieving in target International Normalized Ratio (INR) range at both visits. DNA isolation and genotyping, which included *VKORC1* c.-1639G>A (rs9923231), CYP2C9*2 (p.R144C, rs1799853), and CYP2C9*3 (p.I359L, rs1057910), were performed for each cohort individually as previously described (Galvez et al., 2018; El Rouby et al., 2020). Patients were ≥18 years of age and provided written informed consent for collection of their clinical data and either a venous blood or mouthwash sample for genetic analysis. The clinical studies associated with all sites for the ULLA cohort obtained Ethical and Human Subjects approvals from each organization’s Institutional Review Board. For ULLA data, please contact karnes@pharmacy.arizona.edu.

### Statistical Analyses

Demographic characteristics were compared between IWPC and ULLA cohorts using the tableone R package version 0.12.0 (Yoshida and Bartel, 2020). Prior to analysis, we excluded participants: 1) who did not reach a stable dose, 2) with a weekly dose of over 175 mg or under 7 mg, 3) those with missing gender or age data, 4) with height above 200 cm or under 130 cm, and 5) with weight above 150 kg or under 35 kg, to account for biologically implausible or unlikely values. In the IWPC cohort, a target INR range of 2–3 was implemented. We derived and imputed variables with missing data for each of the datasets using packages and functions available in tidyverse in R version 1.3.0 (Wickham et al., 2019). Allele frequencies for the genetic locations were testing for Hardy-Weinberg Equilibrium using the HWChisq test in the HardyWeinberg package version 1.7.2 (Graffelman, 2015).

In order to address missing data, we imputed or derived missing values following the dataset curation steps described in the Supplementary Materials (Supplementary Methods and Supplementary Table S1). We curated two additional Merged datasets for sensitivity analyses to assess any impact of data curation/imputation on our results. First, we imputed missing values using Multivariate Imputation by Chained Equations with default parameters for diabetes status, statin use, smoking status and aspirin use implemented with the mice package version 3.13.0 (Mera-Gaona et al., 2021). MICE imputes missing values with plausible data values drawn from a distribution specifically designed for each missing datapoint. Second, we performed a complete-case analysis, including only participants with all required data and without imputation. Detailed descriptions of data curation and imputation are available in the Supplementary Materials.

#### Dose Prediction Algorithm Development

**FIGURE 1:**
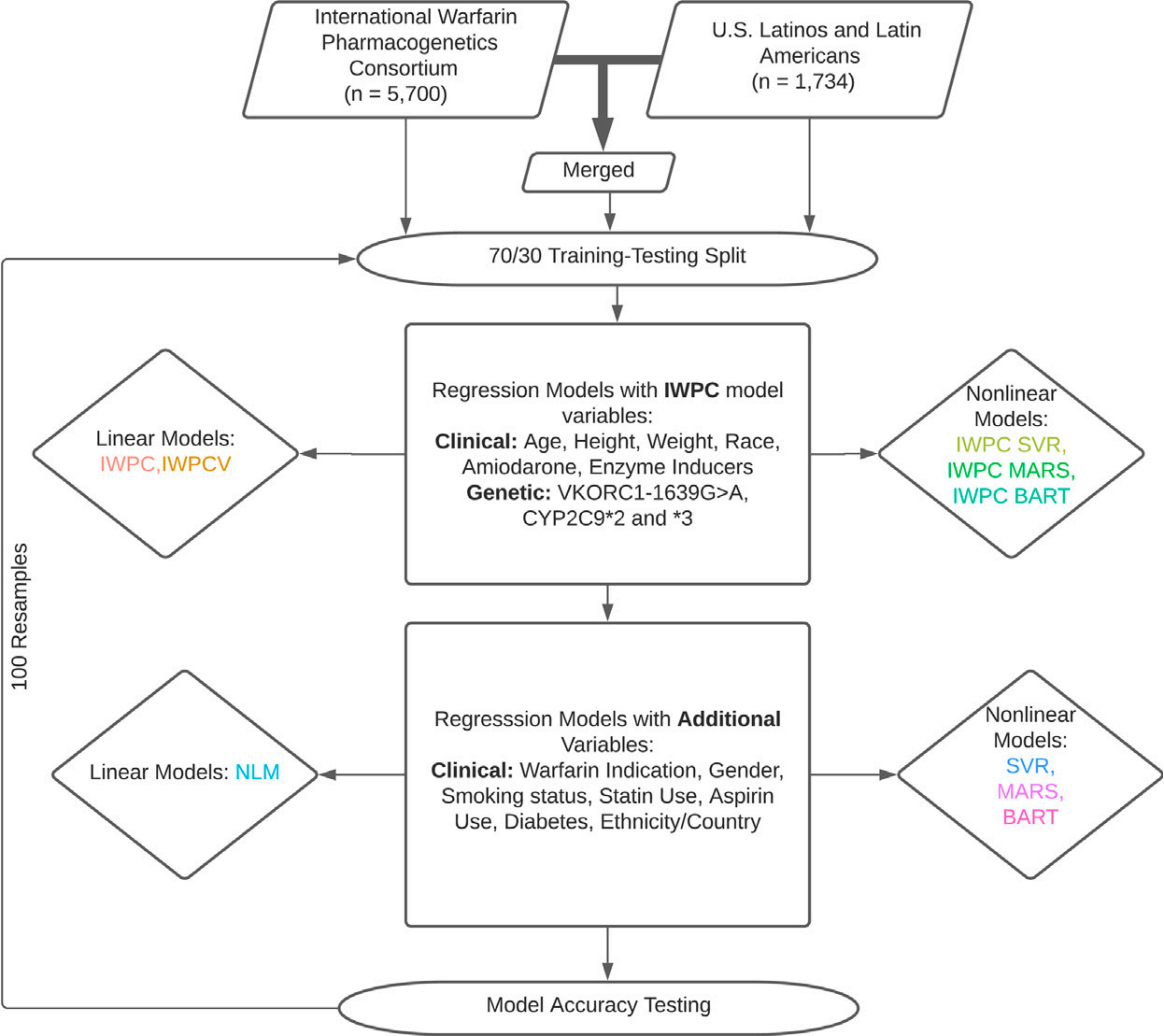
Dose Prediction Algorithm Creation and Testing. International Warfarin Pharmacogenetics Consortium (IWPC) data and data from US Latinos and Latin Americans (ULLA) were used for prediction independently and merged to test a combined sample. Linear and nonlinear models were fit with IWPC model variables and a set of extended variables in addition to IWPC predictors after a 70/30 training-testing split. All models were assessed for their ability to predict dose within 20% of actual. 100 replicates were performed from data splitting to model assessment.

Analyses presented in this study are based on the IWPC model, a novel multiple linear regression model termed the novel linear model (NLM), and three nonlinear regression models: Bayesian Additive Regression Trees (BART), Multivariate Adaptive Regression Splines (MARS), and Support Vector Regression (SVR). Model descriptions are available in Supplementary Methods. First, we reproduced the IWPC analysis with a multiple linear regression model using estimated coefficients derived from the published IWPC model (Figure 1). (International Warfarin Pharmacogenetics Consortium et al., 2009) Second, we predicted dose using the same variables included in the IWPC model but newly trained in the respective cohorts (IWPCV). Thus, the only difference between the IWPC and IWPCV models are the estimated coefficients. We then predicted dose with IWPC variables using the nonlinear methods described above with SVR (IWPC SVR), MARS (IWPC MARS), and BART (IWPC BART). This set of nonlinear models tested the improvement of warfarin dose prediction over IWPC by nonlinear modeling alone. All IWPC models included the variables age, height, weight, genotypes at *CYP2C9* and *VKORC1*, race, amiodarone use, and enzyme inducer use. Next, we created the NLM, including additional predictor variables collected at all study sites (Supplementary Table S1). Finally, we fit another SVR, MARS, and BART using all available variables. The NLM and final three non-linear models (BART, MARS, SVR) included the IWPC variables and the additional variables gender, warfarin indication, statin use, aspirin use, smoking status, history of diabetes, and self-reported ethnicity. In analyses restricted to the ULLA cohort, country of enrollment was also included. All models were fit using the functions outlined below under default parameters using R version 4.0.2 (R Core Team, 2020). We used the *lm* function in the stats R package version 4.0.2 (R Core Team, 2020) to fit linear regression models and generate parameter estimates and standard errors, the *bartMachine* function in the bartMachine package version 1.2.5.1 (Kapelner and Bleich, 2016) for BART models, the *train* function in the caret package version 6.0-86 (Kuhn, 2020) using the “earth” method of the earth package version 5.2.0 (Hastie and wrapper, 2020) for MARS models, and the *svm* function in the e1071 package version 1.7-3 (Meyer et al., 2019) for SVR models. Finally, we estimated variable importance with partial R^2^ values of NLM variables with the *rsq.partial* function in the rsq package version 2.1(Zhang, 2020).

#### Dose Prediction Algorithm Assessment

We applied a square root transformation on weekly warfarin dose when fitting all the models. The primary outcome used to assess model performance was the proportion of patients whose predicted dose was within 20% of their actual stable dose, which represents a clinically relevant difference of 1 mg per day (International Warfarin Pharmacogenetics Consortium et al., 2009). Prior to fitting each replicate within each model, we assigned individuals in each cohort to training and testing datasets. We randomly selected, using a simple random sampling method, 70% among the patients as the training cohort to develop dose-prediction algorithms. The remaining 30% of the patients constituted the testing cohort. Models fit using the training dataset were used to predict values in the training and testing datasets. Estimates of mean absolute error (MAE) and the percentage of individuals predicted within 20% of their actual dose for each model were therefore based on both the training and the testing data. The MAE is the average of the absolute value of predicted dose minus the actual dose, and models with lower MAE tend to better predict the warfarin dose (Willmott and Matsuura, 2005). Uncertainty in model performance was derived from a total of 100 replicates including random resampling of training and testing datasets. Based on all 100 replicates analyzed within each of the models, we estimated the mean and corresponding 95% confidence intervals on MAE and the percentage within 20%. We fit a Friedman test to detect differences in median percentage within 20% across all models using the *friedman_test* function in the rstatix R package version 0.6.0 (Kassambara, 2020). Each linear model’s estimates and standard errors were surveyed from the 50th replicate to maintain consistency in the training/testing data. Finally, we used pairwise Wilcoxon signed-rank tests for paired data to examine whether pairs of models differ in their median proportions within 20% of actual and MAE. We implemented Wilcoxon signed-rank tests using the *wilcox_test* function again in the rstatix R package. All pairwise *p*-values were Bonferroni adjusted to correct for multiple comparisons. The R code associated with the project can be found at https://github.com/karneslab/warfarin-machinelearning.

#### Subgroup Dose Prediction

We explored differences in model performance between subgroups based on actual-dose group, race, and country of enrollment for the ULLA cohort. First, we calculated MAE and percentage within 20% by actual-dose groups: high (>49 mg/week), intermediate, and low (≤21 mg/week). Next, we sought to investigate the validity of utilizing a clinical algorithm without pharmacogenetic variation as suggested by the Clinical Pharmacogenetics Implementation Consortium (CPIC) in patients with self-reported African ancestry who do not have genetic information available for CYP2C9*5,*6,*8, and *11 (Johnson et al., 2017). Thus, the IWPC clinical model, which does not include pharmacogenetic variation, was also used to predict dose in subgroup analyses by race and country of enrollment (International Warfarin Pharmacogenetics Consortium et al., 2009). Next, we evaluated percentage of participants predicted within 20% of actual by race groups. Patients self-reported Black or White race, or were imputed as “Mixed/Missing”. Finally, we examined differences in percentage within 20% and MAE by country/territory of enrollment (i.e. Brazil, Colombia, Puerto Rico, and continental United States) for each of the models.

## Results

### Characteristics of Study Populations

Participants were removed from the PharmGKB IWPC dataset (*n* = 651) when they were outside the target INR range, not on a stable dose of warfarin, missing age and gender data, or were outside the range of inclusion for warfarin stable dose, weight, or height, leaving a total of 5,049 participants. In the ULLA cohort, we excluded 23 patients for a total of 1,734 study participants. To form the merged cohort we removed target INR restrictions and thus fewer (*n* = 404) patients were excluded from IWPC due to missing data or outlying dose, for a total of 7,030 warfarin users in the Merged cohort.

The characteristics of the IWPC and ULLA cohorts are outlined in Table 1. The median (interquartile range [IQR]) weekly warfarin dose (mg) was lower in the IWPC cohort (28.00 [95% Confidence Interval (95%CI) 19.25–38.50] mg/week) than the ULLA cohort (30.00 [95%CI 22.50–37.50] mg/week, *p* <0.001). A small minority (2.4%) of participants in IWPC were carriers of two variant *CYP2C9* alleles. The majority (73.6%) of the participants had no variation in CYP2C9*2 or CYP2C9*3. In the ULLA cohort, 2.4% of the population carried two copies of variant *CYP2C9* alleles, while 79.8% had no variation in CYP2C9*2 or CYP2C9*3. The *VKORC1-*1639G>*A* A allele frequency was 51.4% (AA: 32.5%, GA: 35.8%) in the IWPC cohort and 35.2% (AA: 12.5%, GA: 44.9%) in the ULLA. The IWPC cohort included less than 1% participants reporting Hispanic or Latino ancestry. Alternatively, the ULLA cohort by design was composed of 100% Hispanic or Latino reporting individuals. Demographic and genotype characteristics for the Merged cohort can be found in Supplementary Table S2.

**TABLE 1:** Subject Characteristics in IWPC and ULLA cohorts.

Characteristic	IWPC (*n* = 5,049)	ULLA (n = 1,734)	*P*-Value^a^
Age, years (mean (SD))	59.8 (14.5)	59.7 (13.8)	0.917
Height, cm (median [IQR])	166.88 (160.02–176.02)	166.00 (160.00, 172.72)	<0.001
Weight, kg (median [IQR])	75.40 (62.27–89.70)	75.00 (65.00, 85.00)	0.476
Weekly Warfarin Dose, mg (median [IQR])	28.00 (19.25–38.50)	30.00 (22.50, 37.50)	<0.001
*CYP2C9* Diplotype (*n*, [%])^b^			<0.001
*1/*1	3717 (73.6)	1384 (79.8)	
*1/*2	650 (12.9)	198 (11.4)	
*1/*3	450 (8.9)	83 (4.8)	
*2/*2	46 (0.9)	24 (1.4)	
*2/*3	62 (1.2)	13 (0.7)	
*3/*3	16 (0.3)	4 (0.2)	
Missing	108 (2.1)	28 (1.6)	
*VKORC1* -1639 G>A Genotype (*n* [%])^c^			<0.001
GG	1503 (29.8)	729 (42.0)	
AG	1806 (35.8)	778 (44.9)	
AA	1639 (32.5)	217 (12.5)	
Missing	101 (2.0)	10 (0.6)	
Race (n [%])^d^			<0.001
White	2794 (55.3)	1153 (66.5)	
Asian	1527 (30.2)	0 (0.0)	
Black or African American	451 (8.9)	292 (16.8)	
Mixed or Missing^d^	277 (5.5)	289 (16.7)	
Ethnicity (*n* [%])			<0.001
Hispanic or Latino	35 (0.7)	1734 (100.0)	
not Hispanic or Latino	4139 (82.0)	0 (0.0)	
Unknown	875 (17.3)	0 (0.0)	

IWPC indicates International Warfarin Pharmacogenetics Consortium cohort; ULLA, US Latino and Latin American cohort; SD, standard deviation; IQR, interquartile range; cm, centimeters; kg, kilograms; mg, milligrams.

a *p* values were calculated using a chi Square test for categorical variables, ANOVA for continuous variables and Wilcoxon rank sum test for non-normal continuous variables.

b *CYP2C9* alleles *5, *6, *13, *14 were collapsed into *3 and *11 to *2, consistent with Klein et al.

c *VKORC1* 1639 G>A (rs9923231) rs2359612, rs9934438, rs8050894 were used as tagSNPs where rs9923231 was missing.

d Native American race was collapsed into “Mixed or Missing.”

### Comparison of Predictive Algorithms

In the IWPC cohort (*n* = 5,049), the most accurate model in terms of patients predicted within 20% of actual stable dose in the testing data was the novel NLM (47.4%) and the least accurate model was IWPC MARS (45.6%) (Table 2 and Supplementary Table S3, Supplementary Figure S1 in Supplementary Materials). All models with additional variables not contained in the IWPC model increased accurate dosing prediction by approximately one percent over the IWPC model (all *p* < 4.2 × 10^−12^; Supplementary Tables S3, S6). MAEs were similar for all nine models and ranged from 8.25 to 8.45 mg/week.

**TABLE 2:** Comparison of Warfarin Dose Prediction Algorithms by Median Percentage Predicted within 20% of Actual and Mean Absolute Error (MAE) in the IWPC, ULLA, and Merged cohorts.

	IWPC (*n* = 5,049)		ULLA (*n* = 1,734)		Merged (*n* = 7,030)	
Model	Within 20%^a^	MAE (95%CI)^a^	Within 20%^a^	MAE (95%CI)^a^	Within 20%^a^	MAE (95%CI)^a^
IWPC^b^	45.84	8.36 (7.89–8.85)	47.88	8.12 (7.44–8.82)	46.66	8.24 (7.89–8.58)
IWPCV^b^	45.87	8.41 (7.91–8.87)	47.02	8.20 (7.52–8.90)	46.61	8.24 (7.90–8.59)
IWPC SVR^b^	45.81	8.43 (7.93–8.90)	46.54	8.25 (7.55–8.94)	46.80	8.21 (7.86–8.56)
IWPC MARS^b^	45.575	8.44 (7.95–8.91)	47.50	8.17 (7.49–8.88)	46.56	8.27 (7.92–8.61)
IWPC BART^b^	45.45	8.45 (7.95–8.93)	47.31	8.15 (7.46–8.84)	46.28	8.25 (7.90–8.60)
NLM^c^	47.43	8.25 (7.77–8.74)	47.79	8.11 (7.45–8.79)	47.78	8.13 (7.78–8.47)
SVR^c^	47.33	8.29 (7.8–8.785)	47.41	8.22 (7.52–8.93)	47.61	8.11 (7.77–8.46)
MARS^c^	46.70	8.33 (7.85–8.81)	47.31	8.20 (7.52–8.88)	47.18	8.18 (7.84–8.53)
BART^c^	46.90	8.31 (7.84–8.79)	46.92	8.16 (7.47–8.87)	47.46	8.14 (7.79–8.48)

IWPC indicates International Warfarin Pharmacogenetics Consortium cohort, ULLA, US Latino and Latin American cohort, Merged, ULLA plus IWPC, CI, Confidence Interval, IWPCV, IWPC variables, IWPC MARS, IWPC variables in a Multivariate Adaptive Regression Splines, IWPC SVR, IWPC variables in a Support Vector Regression, IWPC BART, IWPC variables in a Bayesian Additive Regression Trees, NLM, Novel Linear Model.

a Estimates of mean absolute error (MAE) and the percentage of individuals predicted within 20% of their actual dose for each model were based on 100 replicates of resampling testing data.

b Models feature the variables age, height, weight, *CYP2C9* diplotype, *VKORC1* genotype, race, amiodarone use, and enzyme inducer use.

c Models feature the same variables as b in addition to warfarin indication, ethnicity, statin use, aspirin use, history of diabetes.

**FIGURE 2:**
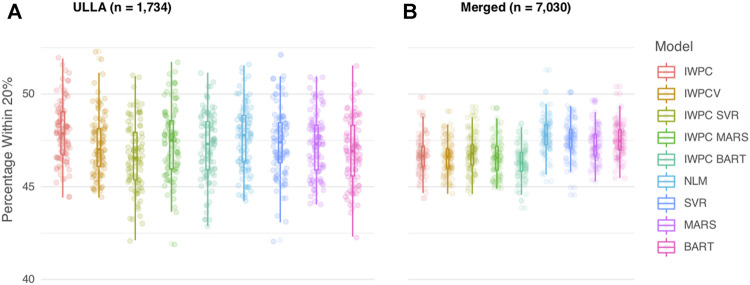
Comparison of Warfarin Dose Prediction Algorithms in the ULLA and Merged cohorts. Proportion of patients predicted within 20% of their actual dose is plotted in the **(A)** US Latinos and Latin Americans (ULLA) cohort and **(B)** Merged cohort containing both ULLA and IWPC cohorts. The boxplot visualizes five summary statistics (the median, 25 and 75% quartiles and two whiskers at 1.5* Interquartile Range). The points indicate the proportion of patients predicted within 20% at each of the 100 rounds of resampling. Models feature IWPC variables or IWPC variables in addition to new predictors. IWPC indicates International Warfarin Pharmacogenetics Consortium model, Merged, IWPC cohort plus ULLA cohort, IWPCV, IWPC variables, IWPC MARS, IWPC variables in a Multivariate Adaptive Regression Splines, IWPC SVR, IWPC variables in a Support Vector Regression, IWPC BART, IWPC variables in a Bayesian Additive Regression Trees, NLM, Novel Linear Model. From left to right, the first five models, IWPC, IWPCV, IWPC_SVR, IWPC_MARS, and IWPC_BART feature the clinical variables age, height, weight, race, enzyme inducer user, amiodarone use and the genetic variables *CYP2C9* Diplotype and *VKORC1*-1639G>A Genotype, the next four models, NLM, SVR, MARS and BART feature the additional variables gender, ethnicity, statin use, aspirin use, history of diabetes, warfarin indication, the last model features only the clinical variables from the first set. IWPC indicates International Warfarin Pharmacogenetics Consortium model, IWPCV, IWPC variables, IWPC MARS, IWPC variables in a Multivariate Adaptive Regression Splines, IWPC SVR, IWPC variables in a Support Vector Regression, IWPC BART, IWPC variables in a Bayesian Additive Regression Trees, NLM, Novel Linear Model, Clinical, the IWPC Clinical model.

In the ULLA cohort (*n* = 1,734), all models performed similarly (Figure 2A, Table 2 and Supplementary Table S4). IWPC predicted 47.9% of the population within 20% of actual dose compared to 47.0% for IWPCV (*p* < 4.28 × 10^−6^, Supplementary Tables S4, S6). While the NLM model had the lowest MAE 8.11 (7.45–8.79), all model MAEs were similar ranging from 8.11 to 8.25 mg/week. The median percentage of participants predicted within 20% in all models fit in the ULLA cohort differed by ∼1%.

In the Merged cohort (*n* = 7,030), the NLM model was the most accurate in this population with 47.8% of the population predicted within 20% of actual dose. All models with additional variables not contained in the IWPC model increased accurate dosing prediction by ∼1% (all *p <* 2.2 × 10^−10^) in the testing data (Figure 2B, Table 2, Supplementary Tables S5, S6). MAEs were similar for all nine models and ranged from 8.11 to 8.27 mg/week. In sensitivity analyses, our results were robust to alternative imputation methods and complete case analysis (Supplementary Results and Supplementary Tables S7, S8).

#### Comparison of Predictive Algorithms by Actual-Dose Groups

**FIGURE 3:**
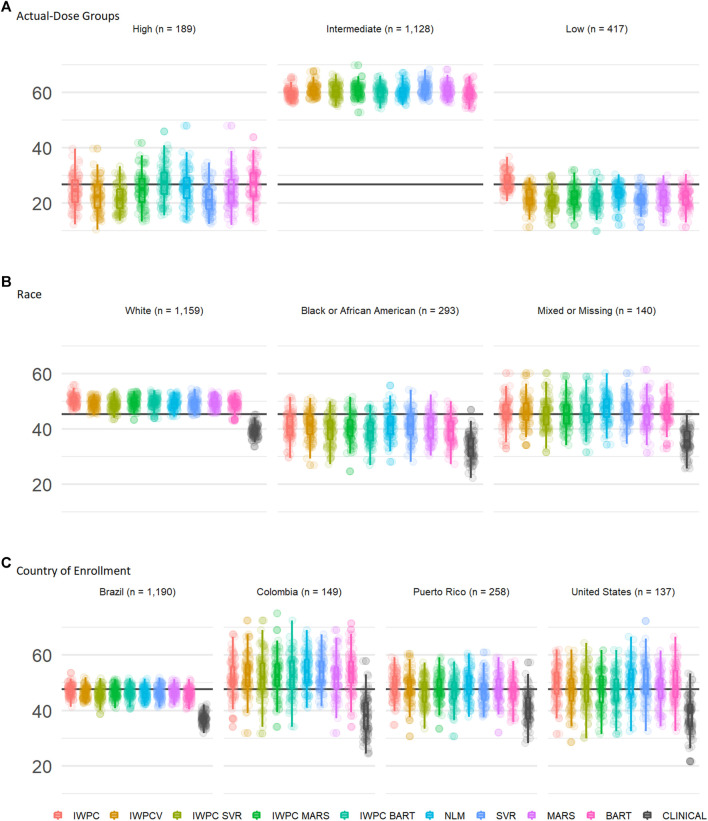
Subgroup Comparisons of Warfarin Dose Prediction Algorithms in the ULLA cohort. Proportion of patients predicted within 20% of their actual dose in the US Latinos and Latin Americans (ULLA) cohort by **(A)** actual-dose group, **(B)** race group, and **(C)** country of enrollment. The boxplot visualizes five summary statistics (the median, 25 and 75% quartiles and two whiskers at 1.5* Interquartile Range). The points indicate the proportion of patients predicted within 20% at each of the 100 rounds of resampling. The horizontal line indicates the median percentage predicted within 20% across all participants. From left to right, the first five models, IWPC, IWPCV, IWPC_SVR, IWPC_MARS, and IWPC_BART feature the clinical variables age, height, weight, race, enzyme inducer user, amiodarone use and the genetic variables *CYP2C9* Diplotype and *VKORC1-*1639G>A Genotype, the next four models, NLM, SVR, MARS and BART feature the additional variables gender, ethnicity, statin use, aspirin use, history of diabetes, warfarin indication, the last model features only the clinical variables from the first set. IWPC indicates International Warfarin Pharmacogenetics Consortium model, IWPCV, IWPC variables, IWPC MARS, IWPC variables in a Multivariate Adaptive Regression Splines, IWPC SVR, IWPC variables in a Support Vector Regression, IWPC BART, IWPC variables in a Bayesian Additive Regression Trees, NLM, Novel Linear Model, Clinical, the IWPC Clinical model.

We assessed performance in the ULLA cohort by actual-dose groups using the nine previously tested models. In the high dose group (weekly warfarin dose >49 mg), both BART models, BART and IWPC_BART, outperformed IWPC (*p <* 1.3 × 10^−4^, Figure 3A and Supplementary Tables S9, S10), while in the low dose group (≤21 mg/week), the IWPC model outperformed all other models (*p <* 1.5 × 10^−15^). In the intermediate group, all models perform similarly and systematically better than in other dose-groups.

#### Comparison of Predictive Algorithms by Race

We assessed performance in the ULLA cohort by self-reported race group using the nine previously tested models alongside the IWPC clinical model, which excludes pharmacogenetic variants(International Warfarin Pharmacogenetics Consortium et al., 2009). In all three race groups, all models outperformed the clinical algorithm by at least 5% (all *p* < 0.001; Figure 3B, Table 3, and Supplementary Table S11). Apart from the clinical model, all models performed similarly in ULLA White and Black race groups. The NLM model outperformed IWPC in the Mixed or Missing race group (*p* = 3.64 × 10^−4^, Supplementary Table S11). The overall mean percentage of patients predicted within 20% of actual dose was highest in the White ULLA race group (49.1%) compared with the Mixed or Missing ULLA race group (45.4%) and the Black ULLA race group (40.0%).

**TABLE 3:** Model comparisons by race data in the ULLA cohort (*n* = 1,734).

	White (*n* = 1,153)		Black (*n* = 292)		Mixed or Missing (*n* = 289)	
Model	Within 20%^a^	MAE (95%CI)^a^	Within 20%^a^	MAE (95%CI)^a^	Within 20%^a^	MAE (95%CI)^a^
IWPC^b^	50.26	7.86 (7.05–8.68)	41.08	8.89 (7.36–10.42)	45.85	8.55 (6.52–10.57)
IWPCV^b^	49.09	7.93 (7.1–8.76)	41.19	8.86 (7.4–10.32)	46.11	8.60 (6.56–10.64)
IWPC SVR^b^	48.78	7.93 (7.09–8.76)	39.73	9.07 (7.59–10.55)	45.19	8.65 (6.60–10.69)
IWPC MARS^b^	49.64	7.88 (7.05–8.71)	40.48	8.99 (7.52–10.46)	45.54	8.60 (6.56–10.64)
IWPC BART^b^	49.62	7.80 (6.99–8.62)	39.04	9.10 (7.60–10.60)	45.60	8.67 (6.57–10.77)
NLM^c^	49.21	7.83 (7.01–8.65)	41.56	8.89 (7.43–10.35)	47.50	8.49 (6.41–10.57)
SVR^c^	49.08	7.92 (7.09–8.75)	41.37	9.07 (7.62–10.52)	46.66	8.55 (6.47–10.63)
MARS^c^	49.48	7.87 (7.04–8.71)	40.51	8.97 (7.50–10.44)	45.37	8.66 (6.62–10.70)
BART^c^	49.05	7.81 (7.00–8.62)	39.30	9.15 (7.67–10.63)	46.12	8.67 (6.54–10.79)
CLINICAL^d^	39.30	9.74 (8.79–10.7)	33.33	10.00 (8.47–11.52)	35.77	10.39 (8.19–12.6)

ULLA indicates US Latino and Latin American warfarin users cohort; CI, Confidence Interval; IWPC, International Warfarin Pharmacogenetics Consortium model; IWPCV, IWPC variables; IWPC MARS, IWPC variables in a Multivariate Adaptive Regression Splines; IWPC SVR, IWPC variables in a Support Vector Regression; IWPC BART, IWPC variables in a Bayesian Additive Regression Trees; NLM, Novel Linear Model.

a Estimates of mean absolute error (MAE) and the percentage of individuals predicted within 20% of their actual dose for each model were based on 100 replicates of resampling 30% testing data.

b Models feature the variables age, height, weight, race, amiodarone use, and enzyme inducer use and genetic variables *CYP2C9* diplotype, *VKORC1* genotype.

^c^Models feature the same variables as b in addition to warfarin indication, ethnicity, statin use, aspirin use, history of diabetes.

d Model features the clinical variables only from b.

#### Comparison of Predictive Algorithms by Country of Enrollment

We assessed performance in the ULLA cohort by country/territory of enrollment using the nine previously tested models alongside the IWPC clinical model, which excludes pharmacogenetic variants (International Warfarin Pharmacogenetics Consortium et al., 2009). In all four national groups, all models outperformed the clinical algorithm by at least 5%, in some cases up to 15% (all *p* < 0.001; Figure 3C and Supplementary Tables S11, S12). The overall mean percentage predicted within 20% of actual dose was highest in the Colombian cohort (51.9%) compared with the continental United States (48.5%), Puerto Rico (47.4%), and Brazil (46.0%).

### Assessment of Linear Models in the Merged Cohort

Parameter estimates, standard errors, and R^2^ values were similar across IWPC, IWPCV, and NLM models (Table 4). For the IWPCV and NLM models that included ULLA patients in their training datasets, we observed similar parameter estimates for pharmacogenetic variable effects relative to the IWPC model. For example, the estimates for the CYP2C9 *1/*2 diplotype ranged from −0.52 ± 0.04 in IWPC to −0.41 ± 0.04 (IWPCV) and to −0.42 ± 0.04 (NLM). In all instances, differences in betas were not outside the confidence intervals of each model. Among the additional variables not contained in the IWPC model, valve replacement indication ( $\hat{\beta }$ = 0.34 ± 0.04, *p* = 7.67 × 10^−16^), deep vein thrombosis indication ( $\hat{\beta }$ = 0.24 ± 0.05, *p* = 1.50 × 10^−7^), history of diabetes ( $\hat{\beta }$ = 0.18 ± 0.04, *p* = 0.001), smoking status ( $\hat{\beta }$ = 0.27 ± 0.04, *p* = 3.28 × 10^−5^), gender ( $\hat{\beta }$ = 0.11 ± 0.03, *p* = 0.0007), unknown ethnicity ( $\hat{\beta }$ = −0.18 ± 0.05, *p* = 0.0001), and statin use ( $\hat{\beta }$ = −0.1 ± 0.04, *p* = 0.007) were associated with warfarin stable dose in the NLM. Hispanic/Latino ethnicity was not significantly associated with warfarin dose ( $\hat{\beta }$ = −0.07 ± 0.04, *p* = 0.1). Partial R^2^ values were consistent for all variables that were included in all three models. Among the additional variables not contained in the IWPC model, we observed that warfarin indication (R^2^ = 0.03) had the highest partial R^2^ value of the additional variables.

**TABLE 4:** Partial R^2^ values, parameter estimates with standard errors, and *p*-values of the 50th replicate of models trained in the Merged cohort (*n* = 7,030).

	IWPC			IWPCV			NLM		
Model Variable	R^2^	***β*** ^ ± SE	*p* ^a^	R^2^	***β*** ^ ± SE	*p* ^a^	R^2^	***β*** ^ ± SE	*p* ^a^
Intercept	-	5.6 ± 0.27	1.11 × 10^−93^	-	5.02 ± 0.27	2.87 × 10^−76^	-	4.12 ± 0.33	4.92 × 10^−35^
Age	0.12	−0.26 ± 0.01	8.82 × 10^−151^	0.12	−0.24 ± 0.01	3.24 × 10^−133^	0.09	−0.21 ± 0.01	6.65 × 10^−88^
Height	0.02	0.01 ± 0	1.73 × 10^−07^	0.02	0.01 ± 0	5.17 × 10^−12^	0.02	0.01 ± 0	1.42 × 10^−14^
Weight	0.04	0.01 ± 0	1.14 × 10^−41^	0.04	0.01 ± 0	3.27 × 10^−37^	0.04	0.01 ± 0	1.71 × 10^−38^
*CYP2C9* ^b^	0.11	-	-	0.11	-	-	0.11	-	-
*1/*2	-	−0.52 ± 0.04	4.75 × 10^−33^	-	−0.41 ± 0.04	2.5 × 10^−21^	-	−0.42 ± 0.04	9.25 × 10^−23^
*1/*3	-	−0.94 ± 0.05	9.18 × 10^−72^	-	−0.85 ± 0.05	2.02 × 10^−59^	-	−0.86 ± 0.05	1.16 × 10^−62^
*2/*2	-	−1.06 ± 0.14	1.49 × 10^−13^	-	−0.97 ± 0.14	1.53 × 10^−11^	-	−0.96 ± 0.14	1.22 × 10^−11^
*2/*3	-	−1.92 ± 0.13	1.63 × 10^−49^	-	−1.54 ± 0.13	9.94 × 10^−33^	-	−1.56 ± 0.13	2.76 × 10^−34^
*3/*3	-	−2.33 ± 0.29	2.73 × 10^−15^	-	-2.24 ± 0.29	3.46 × 10^−14^	-	-2.24 ± 0.29	1.24 × 10^−14^
Missing	-	−0.22 ± 0.1	0.0293	-	−0.2 ± 0.1	0.047	-	−0.23 ± 0.1	0.0214
*VKORC1* ^c^	0.23	-	-	0.23	-	-	0.23	-	-
A/G		−0.87 ± 0.03	1.77 × 10^−139^	-	−0.8 ± 0.03	1.2 × 10^−119^	-	−0.79 ± 0.03	2.25 × 10^−121^
A/A		−1.7 ± 0.04	2.03 × 10^−279^	-	−1.62 ± 0.04	2.34 × 10^−256^	-	−1.61 ± 0.04	3.87 × 10^−260^
Missing		−0.49 ± 0.12	2.7 × 10^−05^	-	−0.34 ± 0.12	0.00299	-	−0.38 ± 0.11	0.00103
Race	0.01	-	-	0.01	-	-	0.02	-	-
Asian		−0.11 ± 0.05	0.0231	-	−0.1 ± 0.05	0.0423	-	−0.16 ± 0.05	0.00263
Black or African American		−0.28 ± 0.05	1.75 × 10^−08^	-	−0.16 ± 0.05	0.000908	-	−0.21 ± 0.05	2.51 × 10^−5^
Mixed or Missing^d^		−0.1 ± 0.05	0.0457	-	−0.07 ± 0.05	0.152	-	0.02 ± 0.06	0.71
Enzyme Inducer Use	0.02	1.18 ± 0.13	8.37 × 10^−21^	0.02	0.85 ± 0.13	1.83 × 10^−11^	0.02	0.78 ± 0.12	2.99 × 10^−10^
Amiodarone Use	0.04	−0.55 ± 0.04	2.16 × 10^−37^	0.04	−0.54 ± 0.04	1.42 × 10^−36^	0.03	−0.45 ± 0.05	2.28 × 10^−21^
Ethnicity	-	-	-	-	-	-	0.01	-	-
Hispanic/Latino	-	-	-	-	-	-	-	−0.07 ± 0.04	0.117
Unknown	-	-	-	-	-	-	-	−0.18 ± 0.05	0.000133
Gender (female)	-	-	-	-	-	-	0.01	0.11 ± 0.03	0.000701
Statin Use	-	-	-	-	-	-	0.01	−0.1 ± 0.04	0.00794
Aspirin Use	-	-	-	-	-	-	0.01	−0.07 ± 0.04	0.117
Indication	-	-	-	-	-	-	0.03	-	-
DVT/PE	-	-	-	-	-	-		0.24 ± 0.05	1.5 × 10^−7^
TIA	-	-	-	-	-	-		−0.03 ± 0.08	0.689
Valve	-	-	-	-	-	-		0.34 ± 0.04	7.67 × 10^−16^
Other	-	-	-	-	-	-		-0.01 ± 0.04	0.873
Diabetes	-	-	-	-	-	-	0.01	0.18 ± 0.04	3.28 × 10^−5^
Smoking status	-	-	-	-	-	-	0.01	0.27 ± 0.05	4.24 × 10^−7^
Total R^2^	47.03			47.03			48.55

IWPC indicates International Warfarin Pharmacogenetics Consortium model; IWPCV, the same variables as IWPC in a new model; NLM, Novel Linear Model including the additional predictors: statin use, aspirin use, warfarin indication, ethnicity, history of diabetes; SE, Standard Error; DVT, Deep Vein Thrombosis; PE, Pulmonary Embolism; TIA, Transient Attack; AFIB, Atrial Fibrillation

a *p*-values determined by the lm function in R.

b *CYP2C9* Diplotypes *5, *6, *13, *14 collapsed into *1/*3 and *11 to *1/*2.

c VKORC1 1639 G>A (rs9923231) rs2359612, rs9934438, rs8050894 were used as proxies where rs9923231 was missing.

d Native American race was collapsed into “Mixed or Missing”.

## Discussion

This study combined a large US Latino and Latin American cohort with IWPC data, constituting the largest available cohort for modelling stable warfarin dose in Hispanic and Latino patients. We found that IWPC models were accurate when applied to both our ULLA population and a combined cohort of ethnically diverse patients. We found limited evidence that nonlinear models significantly improve prediction of warfarin dose compared to linear models in any cohort in this analysis. Inclusion of additional predictor variables resulted in a small but significant improvement of prediction of warfarin dose relative to the published IWPC model. Specifically, the inclusion of warfarin indication, smoking status, diabetes, statin use, and gender informed warfarin dose prediction above that of the IWPC and IWPCV models. These results suggest that several important variables are not currently being captured by commonly used warfarin dose prediction algorithms. In care settings where warfarin dose algorithms are implemented, these data, which are routinely collected in electronic health record systems and in clinical assessments of warfarin users, should be accurate and readily available for improvement of algorithm accuracy.

In our study, nonlinear models did not out-perform linear regression models in our Latino/Latin American cohort, an observation that is inconsistent with some previous literature in other populations. One study used the IWPC cohort to model warfarin dose using nonlinear models, finding increased prediction accuracy with nonlinear models in under 400 Italian warfarin users (Liu et al., 2015). Another study investigated machine learning for predicting warfarin dose in a small Caribbean Hispanic population with similar results (Roche-Lima et al., 2020). However, neither study compared new models to the IWPC model. Another study observed improved warfarin dose prediction over IWPC with a nonlinear model using seven additional variables as used in our analysis, but no comparisons were made between linear and nonlinear models in the same cohort(Grossi et al., 2014). While some previous literature suggests nonlinear models may outperform multiple linear regression methods when used to predict warfarin dose, our observations suggest that linear models perform similarly to nonlinear models in diverse populations including a high number of ULLA participants.

Our results also demonstrate the robustness of the IWPC model in a diverse patient population and in ULLA populations. Overall, these results suggest the validity of utilizing IWPC algorithms in patients with Latino/Latin American ethnicity consistent with CPIC guideline recommendations (Johnson et al., 2017). Consistent with this observation, Latino/Latin American ethnicity was not associated with stable warfarin dose in our novel linear model. The median weekly dose was higher in the ULLA cohort, which may have been due to differential allele frequencies in important pharmacogenes (International Warfarin Pharmacogenetics Consortium et al., 2009). The *VKORC1*-1639 A allele frequency was 51.4% in the IWPC cohort and just 31.3% in the ULLA, and the percentage of patients carrying a CYP2C9*2 or *3 variant was lower in ULLA (20.4%) than IWPC (26.4%). These observations are consistent with previous literature reporting frequency of these variants in Hispanic and Latino populations (Kaye et al., 2017).

Subgroup analysis of actual-dose groups in our ULLA cohort showed a similar story as the overall results: IWPC performs as well as newly developed and trained models. In the low dose group, there was a stark decline in model accuracy as compared to the IWPC model. This result suggests that initial estimation of dose-groups may facilitate model choice for dose prediction. Latino individuals requiring low doses may benefit the most from dose prediction with the IWPC model.

In our ULLA cohort, the IWPC model performed as well numerous models trained in this cohort. This result may be due to a high rate of European admixture in our ULLA cohort, which we were not able to evaluate since sufficient genome-wide data was not available on all ULLA participants. In subgroup analysis of country/territory of enrollment, the Colombian cohort showed a marked advantage in prediction. This improved performance could be due to a larger proportion of European ancestry in this cohort relative to, for instance, our Brazilian cohort which had a higher proportion of self-reported Black participants (Wang et al., 2008; Salzano and Sans, 2014). It is also probable that more Latin American participants are included in the publicly available IWPC dataset than are indicated by the Hispanic/Latino ethnicity variable. Multiple data contributors in Latin America were listed in this effort, but only 1 percent of patients were considered Hispanic/Latino and this small number of participants were from multiple sites (International Warfarin Pharmacogenetics Consortium et al., 2009). Our observation that all models had lower accuracy in ULLA participants who self-reported Black or African American race reinforces previous work indicating that IWPC models perform poorly in individuals with African ancestry, in part due to the disregard for CYP2C9*5, *6, *8, and *11 alleles(Kimmel et al., 2013; Drozda et al., 2015).

Current CPIC guidelines for pharmacogenetic-guided warfarin dosing recommend different approaches for patients reporting African ancestry(Johnson et al., 2017). This is largely based on observations from the Clarification of Optimal Anticoagulation through Genetics (COAG) trial, which limited *CYP2C9* genotyping to the *2 and *3 alleles and showed that Black patients spent less time in the therapeutic range in the pharmacogenetics-guided group than in the clinically-guided group(Kimmel et al., 2013; French et al., 2016). Subsequent analysis showed that not accounting for *CYP2C9* variants common in people with African ancestry lead to significant over-dosing in Black patients. While our analyses suggest that increasing African ancestry leads to poor algorithm performance, our results also suggests that the IWPC clinical model underperforms for ULLA patients with self-reported black race. ULLA patients who report black race might be at risk of overdosing by disregarding genetic information in warfarin dosing, regardless of the presence of *CYP* variants of high predictive value in individuals of African ancestry. This observation may be due to a lower proportion of African ancestry in Black ULLA participants relative to African Americans from the COAG trial. Our observations in specific race and country/territory groups should be interpreted with caution as sample sizes are small after implementing a 70/30 training-testing split.

There are several limitations that are worthy of mention in this study. We were limited by the use of retrospective data to the variables that were included in the publicly available IWPC dataset. Since the publication of IWPC, a number of studies have reported additional warfarin dose predictor variables that might be included in future studies (Asiimwe et al., 2020; Roche-Lima et al., 2020). While we chose to focus on IWPC, other algorithms such as the Gage et al. algorithm might also have been tested. However, the dataset used to derive the Gage et al. algorithm was included in the IWPC dataset and both IWPC and Gage et al. algorithms have been shown to perform similarly across populations (Shin and Cao, 2011). Pharmacogenetic information used in this analysis was also limited to CYP2C9*2,*3 and *VKORC1-*1639G>A, which are variants identified in studies of primarily White populations. CYP2C9 *5,*6, *8, and *11 are important in the prediction of warfarin dose in Black or African patients and additional variants in *CALU,* the CYP2C cluster (e.g. rs12777823), and *GGCX* have been shown to affect warfarin dose (Wadelius et al., 2005; Voora et al., 2010). Furthermore, studies have identified the NQ O 1*2 (p. P187S; rs1800566) variant as a contributor to warfarin dose variation in Hispanic and Latino patients, and this genotype information was not available in the IWPC dataset (Bress et al., 2012; El Rouby et al., 2020). Data from a pharmacogenomic or genome-wide SNP platform would likely provide additional information useful in warfarin dose prediction, including additional CYP variants that are not biased by low MAFs in the discovery population and admixture proportions, both of which have been identified as important warfarin dose prediction variables(Hernandez et al., 2020). Apart from genetic variation, other potential sources of warfarin dose variability, including medication adherence data and environmental exposures such as vitamin K intake, were not available for this analysis.

## Conclusion

In this systematic comparison of nine models, classic linear regression models remained advantageous compared to nonlinear models with respect to prediction accuracy of therapeutic warfarin dose in a large diverse cohort as well as a Hispanic/Latino cohort alone. Our results suggest that the inclusion of additional predictor variables, beyond those used in the IWPC model but often collected during warfarin treatment, may improve accuracy of warfarin stable dose algorithms. Our results also suggest that the IWPC model is accurate for stable dose prediction in populations with Hispanic/Latino ethnicity, with the possible exception of Afro-Latino warfarin users. This result warrants further exploration in additional Hispanic/Latino cohorts with careful consideration for race. Furthermore, our results indicate that the IWPC clinical model performs poorly relative to all other algorithms tested for US Latino and Latin American patients, regardless of whether they report African ancestry.

## Data Availability

The datasets analyzed in this study are not publicly available due to privacy/ethical restrictions. Requests to access these datasets should be directed to karnes@pharmacy.arizona.edu.
