# Supplementary material for: Machine Learning for Prediction of Stable Warfarin Dose in US Latinos and Latin Americans
Source: Front Pharmacol. 2021 Oct 29;12:749786. doi: 10.3389/fphar.2021.749786 (PMC8585774; doi:10.3389/fphar.2021.749786)
Supplement: Supplementary file 1 [file DataSheet1.docx]

**Supplementary Methods**

### Dataset Curation

We derived and imputed variables with missing data for each of the datasets using packages and functions available in tidyverse in R version 1.3.0.(Wickham et al., 2019) The following variables were derived following IWPC (Supplementary Appendix therein): height, weight, race, enzyme inducer use, amiodarone use, and genotypes at *CYP2C9* and *VKORC1-1639 A>G* (**Table S1**).(International Warfarin Pharmacogenetics Consortium et al., 2009) Missing values of amiodarone use were imputed as no amiodarone use. Enzyme-inducer use was derived by adding exposures from phenytoin, carbamazepine, rifampin and rifampicin in which patient drug use was dichotomized as taking the drug or not. We derived the final *CYP2C9* genotype by compounding *1/*11 with *1/*2 as well as *1/*5 and *1/*6 with *1/*3, as these variants are rarer and have similar metabolizing effects on warfarin.(Asiimwe et al., 2020) VKORC1 was imputed based on race and genotype at rs23599612, rs9934438 and rs8050894, as described in the IWPC supplementary materials.(International Warfarin Pharmacogenetics Consortium et al., 2009) Where both race and self-reported race were missing, a new category “Mixed or Missing” was formed. As in the original IWPC manuscript, height and weight were imputed by the mean at the same gender and race combination. Additional variables included in the IWPC dataset were analyzed even though they were not part of the final model from the IWPC publication. Aspirin use, statin use, smoking status, and history of diabetes were considered no drug use/no history of if missing (**Table S1**). Statins included: simvastatin, rosuvastatin, atorvastatin, fluvastatin, lovastatin, pravastatin, cerivastatin. Hispanic and Latino or non-Hispanic or Latino ethnicities were imputed using self-reported ethnicity. Indications for warfarin were condensed into Atrial Fibrillation, Deep Vein Thrombosis or Pulmonary Embolism, Stroke, Heart valve replacement or other.

### Model Descriptions

Multiple linear regressions are the standardized models used in warfarin dose prediction. In linear regression, relationships are modeled in a linear fashion, with parameters estimated by minimizing the errors between observed and predicted values. In addition to the linear assumption, linear regression models assume constant variance across all values of the independent variables, independence observations, and homoscedasticity of the residuals. The three additional nonlinear regression models that were fit in this study were selected based on their superior performance in previous papers.(Liu et al., 2015; Roche-Lima et al., 2020) The Support Vector Regression (SVR) does not depend on gaussian assumptions as linear regression does but is otherwise similar in function. SVR attempts to minimize model coefficients while using prediction errors as constraints to the examined lines.(Awad and Khanna, 2015) Multivariate Adaptive Regression Splines (MARS) are a flexible alternative to linear regression that builds multiple, smaller linear regression model chunks across a range of predictor values.(Friedman, 1991) No assumptions are made about the distributions of the variables in a MARS model. Bayesian Additive Regression Trees (BART) is a Bayesian regression approach that is able to fit interactions and nonlinearities by adding together information from many regression trees.(Chipman et al., 2010)

# **Supplementary Results**

### Basic Characteristics of the Merged Cohort

### A total of 7,030 patients were included in the Merged cohort (**Table S2**). The cohort had a mean (±SD) age of 59.5±14.4 years. The median (IQR) weekly warfarin dose was 28.00 mg/week (CI: 20.00-38.50) mg/week. A majority of the population (75%) had no variation in *CYP2C9*1* and *CYP2C9*2* and 9.0% carried two copies of variant alleles. The frequencies of VKORC1 genotypes, A/A, A/G, and G/G, were 26.7%, 38.3%, and 33.4%, respectively. Our Merged dataset included 25% participants of Latin American ethnicity. Overall, there were differences between the IWPC and ULLA cohorts.

### Sensitivity Analyses

We found our results were consistent when our sensitivity datasets were used to train models for warfarin dose prediction. In the Merged cohort featuring Multivariate Imputation by Chained Equations, patterns of model performance remain the same with extended variable models again outperforming IWPC by <1% (**Table S7**). MAEs were similar for all nine models and ranged from 7.81 - 8.28 mg/week (**Table S7**). Each of the IWPC variable models (i.e. IWPC, IWPCV, IWPC_SVR, IWPC_MARS, IWPC_BART) was used to predict dose in the complete-case dataset. The remaining models were not tested as the missing data were over 50% when including the additional variables. In the complete-case cohort (n = 3,420), where all observations with incomplete data were removed, the accuracy of the IWPC model fell to 45%, from 46% in the Merged cohort (**Table S8**). However, prediction within 20% of actual for the rest of the models remained the same ~47% between the cohorts. Thus, our data indicate that IWPC model performance is improved through imputation, whereas the other models appear to be unaffected.

| Table S1. Dataset Curation and treatment of missing data in the primary analyses. | | |
| --- | --- | --- |
| **Variable** | **Curation^a^** | **Missing Data^b^** |
| Gender | 0 = Male, 1 = Female | - |
| **Race (Reported)** |  | **race = "Mixed or Missing"** |
| **Race (OMB)** | **factored as White, Black or African American, Asian, Mixed or Missing** | **race = Race (Reported)** |
| Ethnicity (Reported) | factored as not Hispanic or Latino, Hispanic or Latino, Unknown | IWPC ethnicity = "Unknown",  ULLA ethnicity = "Hispanic/Latino" |
| Ethnicity (OMB) | factored as not Hispanic or Latino, Hispanic or Latino, Unknown | ethnicity = Ethnicity (Reported) |
| **Age at Consent, years** | **converted to decades** | **removed** |
| **Height, cm** | **-** | **mean height at the same race and gender** |
| **Weight, kg** | **-** | **mean weight at the same race and gender** |
| Primary Indication for Warfarin Treatment | Factored as DVT/PE, AFIB, Valve, TIA, Other where Valve replacement > AFIB > TIA > DVT > Other in patients with multiple indications | Factored as “Other” |
| Diabetes | 0 = no history, 1 = history of diabetes | diabetes = 0 |
| Aspirin | 0 = no use, 2 = use of aspirin | aspirin = 0 |
| Statin | simvastatin, rosuvastatin, atorvastatin, fluvastatin, lovastatin, pravastatin, cerivastatin = use of statin | statin = 0 |
| **Amiodarone (Cordarone)** | **0 = no use, 2 = use of amiodarone** | **amiodarone = 0** |
| **Carbamazepine (Tegretol)** | **cabamazepine, phenytoin, rifampin, rifampicin = use of enyzyme inducer (ei)** | **ei = 0** |
| **Phenytoin (Dilantin)** | **cabamazepine, phenytoin, rifampin, rifampicin = use of enyzyme inducer (ei)** | **ei = 0** |
| **Rifampin or Rifampicin** | **cabamazepine, phenytoin, rifampin, rifampicin = use of enyzyme inducer (ei)** | **ei = 0** |
| **Stable** | **0 = did not reach stable dose, 1 = reached stable dose** | **removed** |
| **Warfarin Dose (mg/week)** | **-** | **removed** |
| Current Smoker | 0 = no, 1 = yes | smoke = 0 |
| ***CYP2C9* diplotypes** | **factored as *1/*1, *1/*2, *1/*3, *2/*3, *3/*3 where *1/*11 was *1/*2 and *1/*5 and *1/*6 was *1/*3,** | **Factored as “Missing”** |
| ***VKORC1* genotype -1639 G>A; rs9923231** | **factored as AA, AG, GG** | **rs2359612, rs9934438, rs8050894 used as proxies; factored as “Missing”** |
| OMB indicates Office of Management and Budget, cm, centimeters, kg, kilograms, DVT, Deep Vein ThrombosisPE, Pulmonary Embolism, AFIB, Atrial Fibrillation, Valve, Valvular Replacement; ei, enzyme inducer; TIA, transient ischemic attack; ULLA, IWPC, | | |

^a^ Curation indicates cleaning data to normalize across datasets. Bolded rows indicate variables imputed or derived following Klein *et al.* (supplemental methods therein)

^b^ Missing Data indicates the data curation performed when data was missing at that variable

| **Table S2. Subject Characteristics in the Merged Cohort (n=7,030) ^a^** | |
| --- | --- |
| **Characteristic** | **Result** |
| **Age, years (mean (SD))** | 59.5 (14.4) |
| **Height, cm (median [IQR])** | 166.88 (160.00-175.01) |
| **Weight, kg (median [IQR])** | 75.00 (63.23-88.60) |
| **Weekly Warfarin Dose, mg (median [IQR])** | 28.00 (20.00-38.50) |
| ***CYP2C9* Diplotype (n, [%])^b^** |  |
| ***1/*1** | 5273 (75.0) |
| ***1/*2** | 895 (12.7) |
| ***2/*2** | 556 (7.9) |
| ***1/*3** | 75 (1.1) |
| ***2/*3** | 73 (1.0) |
| ***3/*3** | 17 (0.2) |
| **Missing** | 141 (2.0) |
| ***VKORC1 1639 A>G* Genotype (n, [%])^c^** |  |
| **GG** | 2348 (33.4) |
| **AG** | 2695 (38.3) |
| **AA** | 1879 (26.7) |
| **Missing** | 108 (1.5) |
| **Race (%)** |  |
| **White** | 4136 (58.8) |
| **Asian** | 1520 (21.6) |
| **Black or African American** | 770 (11.0) |
| **Mixed or Missing^d^** | 604 (8.6) |
| **Ethnicity (%)** |  |
| **Not Hispanic or Latino** | 4245 (60.4) |
| **Hispanic or Latino** | 1776 (25.3) |
| **Unknown** | 1009 (14.4) |
| SD indicates Standard Deviation, IQR, Interquartile Range  ^a^ Merged indicates International Warfarin Pharmacogenetics Consortium data with six cohorts of U.S. Latinos and Latin Americans  ^b^*CYP2C9* alleles *5, *6, *13, *14 collapsed into *1/*3 and *11 to *1/*2  ^c^VKORC1 *1639 A>G* (rs9923231), rs2359612, rs9934438, rs8050894 were used as proxies where rs9923231 was missing  ^d^ Native American race was collapsed into “Mixed or Missing” | |

| **Table S3. Model comparisons between testing and training data in the IWPC cohort (n = 5,049).** | | | | |
| --- | --- | --- | --- | --- |
|  | **Training (n = 3,534)** | | **Testing (n= 1,515)** | |
| **Model** | **Within 20%^a^** | **MAE (95% CI) ^a^** | **Within 20%^a^** | **MAE (95% CI) ^a^** |
| IWPC **^b^** | 46.00 | 8.38 (8.07-8.69) | 45.84 | 8.36 (7.89-8.85) |
| IWPCV **^b^** | 46.12 | 8.37 (8.06-8.67) | 45.87 | 8.41 (7.91-8.87) |
| IWPC SVR **^b^** | 47.56 | 8.19 (7.88-8.50) | 45.81 | 8.43 (7.93-8.90) |
| IWPC MARS **^b^** | 45.89 | 8.39 (8.08-8.69) | 45.58 | 8.44 (7.95-8.91) |
| IWPC BART **^b^** | 46.86 | 8.12 (7.84-8.40) | 45.45 | 8.45 (7.95-8.93) |
| NLM **^c^** | 47.59 | 8.2 (7.91-8.51) | 47.43 | 8.25 (7.77-8.74) |
| SVR **^c^** | 49.46 | 7.97 (7.66-8.27) | 47.33 | 8.29 (7.8-8.785) |
| MARS **^c^** | 47.00 | 8.28 (7.97-8.59) | 46.70 | 8.33 (7.85-8.81) |
| BART **^c^** | 48.47 | 7.93 (7.65-8.22) | 46.90 | 8.31 (7.84-8.79) |
| IWPC, International Warfarin Pharmacogenetics Consortium cohort/model; MAE, mean absolute error; CI, confidence interval; IWPCV, IWPC variables; IWPC MARS, IWPC variables in a Multivariate Adaptive Regression Splines; IWPC SVR, IWPC variables in a Support Vector Regression; IWPC BART, IWPC variables in a Bayesian Additive Regression Trees; NLM, Novel Linear Model  **^a^** Estimates of mean absolute error (MAE) and the percentage of individuals predicted within 20% of their actual dose for each model were based on 100 replicates of resampling testing data.  **^b^** Models feature the variables age, height, weight, race, amiodarone use, and enzyme inducer use and genetic variables *CYP2C9* diplotype, *VKORC1* genotype  **^c^** Models feature the same variables as b in addition to warfarin indication, ethnicity, statin use, aspirin use, history of diabetes | | | | |

| **Table S4. Model comparisons between testing and training data in the ULLA cohort (n = 1,734).** | | | | |
| --- | --- | --- | --- | --- |
|  | **Training (n = 1,214)** | | **Testing (n = 520)** | |
| **Model** | **Within 20% ^a^** | **MAE (95% CI) ^a^** | **Within 20% ^a^** | **MAE (95% CI) ^a^** |
| IWPC **^b^** | 48.02 | 8.18 (7.70-8.64) | 47.88 | 8.12 (7.44-8.82) |
| IWPCV **^b^** | 47.78 | 8.09 (7.63-8.55) | 47.02 | 8.20 (7.52-8.90) |
| IWPC SVR **^b^** | 50.16 | 7.85 (7.38-8.32) | 46.54 | 8.25 (7.55-8.94) |
| IWPC MARS **^b^** | 48.27 | 8.01 (7.55-8.47) | 47.50 | 8.17 (7.49-8.88) |
| IWPC BART **^b^** | 49.59 | 7.69 (7.26-8.12) | 47.31 | 8.15 (7.46-8.84) |
| NLM **^c^** | 48.48 | 7.94 (7.48-8.39) | 47.79 | 8.11 (7.45-8.79) |
| SVR **^c^** | 51.98 | 7.66 (7.20-8.13) | 47.41 | 8.22 (7.52-8.93) |
| MARS **^c^** | 48.39 | 8.02 (7.55-8.49) | 47.31 | 8.20 (7.52-8.88) |
| BART **^c^** | 50.25 | 7.53 (7.11-7.96) | 46.92 | 8.16 (7.47-8.87) |
| ULLA, U.S. Latino and Latin American Warfarin User Cohort; MAE, mean absolute error; CI, confidence interval; IWPC, International Warfarin Pharmacogenetics Consortium model; IWPCV, IWPC variables; IWPC MARS, IWPC variables in a Multivariate Adaptive Regression Splines; IWPC SVR, IWPC variables in a Support Vector Regression; IWPC BART, IWPC variables in a Bayesian Additive Regression Trees; NLM, Novel Linear Model  **^a^** Estimates of mean absolute error (MAE) and the percentage of individuals predicted within 20% of their actual dose for each model were based on 100 replicates of resampling testing data.  **^b^** Models feature the variables age, height, weight, race, amiodarone use, and enzyme inducer use and genetic variables *CYP2C9* diplotype, *VKORC1* genotype  **^c^** Models feature the same variables as b in addition to warfarin indication, ethnicity, statin use, aspirin use, history of diabetes | | | | |

| **Table S5. Model comparisons among testing and training data in the Merged cohort (n = 7,030).** | | | | |
| --- | --- | --- | --- | --- |
|  | **Training (n = 4,639)** | | **Testing (n = 1,988)** | |
| **Model** | **Within 20% ^a^** | **MAE (95% CI) ^a^** | **Within 20% ^a^** | **MAE (95% CI) ^a^** |
| IWPC **^b^** | 46.70 | 8.26 (8.03-8.49) | 46.66 | 8.24 (7.89-8.58) |
| IWPCV **^b^** | 46.71 | 8.23 (8.00-8.47) | 46.61 | 8.24 (7.90-8.59) |
| IWPC SVR **^b^** | 48.18 | 8.05 (7.81-8.28) | 46.80 | 8.21 (7.86-8.56) |
| IWPC MARS **^b^** | 46.64 | 8.24 (8.00-8.47) | 46.56 | 8.27 (7.92-8.61) |
| IWPC BART **^b^** | 47.41 | 8.05 (7.83-8.28) | 46.28 | 8.25 (7.90-8.60) |
| NLM **^c^** | 48.04 | 8.10 (7.87-8.33) | 47.78 | 8.13 (7.78-8.47) |
| SVR **^c^** | 49.75 | 7.85 (7.62-8.08) | 47.61 | 8.11 (7.77-8.46) |
| MARS **^c^** | 47.59 | 8.15 (7.92-8.37) | 47.18 | 8.18 (7.84-8.53) |
| BART **^c^** | 48.69 | 7.91 (7.69-8.12) | 47.46 | 8.14 (7.79-8.48) |
| MAE, mean absolute error; CI, confidence interval; IWPC, International Warfarin Pharmacogenetics Consortium model; IWPCV, IWPC variables; IWPC MARS, IWPC variables in a Multivariate Adaptive Regression Splines; IWPC SVR, IWPC variables in a Support Vector Regression; IWPC BART, IWPC variables in a Bayesian Additive Regression Trees; NLM, Novel Linear Model  **^a^** Estimates of mean absolute error (MAE) and the percentage of individuals predicted within 20% of their actual dose for each model were based on 100 replicates of resampling testing data.  **^b^** Models feature the variables age, height, weight, race, amiodarone use, and enzyme inducer use and genetic variables *CYP2C9* diplotype, *VKORC1* genotype  **^c^** Models feature the same variables as b in addition to warfarin indication, ethnicity, statin use, aspirin use, history of diabetes | | | | |

| **Table S6. Pairwise Bonferroni Adjusted *P*-values of Median Percentage Predicted Within 20% of Actual Dose in Each Cohort ^a^** | | | | |
| --- | --- | --- | --- | --- |
| **Model 1** | **Model 2** | **IWPC^b^** | **ULLA^b^** | **Merged^b^** |
| **IWPC** | **IWPCV** | 1 | 4.28x10^-6^ | 0.329 |
| **IWPC** | **IWPC_SVR** | 1 | 9.79x10^-12^ | 1 |
| **IWPC** | **IWPC_MARS** | 4.21x10^-4^ | 0.012 | 1 |
| **IWPC** | **IWPC_BART** | 0.055 | 6.05x10^-5^ | 1.44x10^-5^ |
| **IWPC** | **NLM** | 1.46x10^-16^ | 0.551 | 1.43x10^-15^ |
| **IWPC** | **SVR** | 2.96x10^-16^ | 0.011 | 7.49x10^-14^ |
| **IWPC** | **MARS** | 4.14x10^-12^ | 7.27x10^-4^ | 2.16x10^-10^ |
| **IWPC** | **BART** | 4.68x10^-15^ | 1.36x10^-7^ | 2.05x10^-13^ |
| **IWPCV** | **IWPC_SVR** | 1 | 8.35x10^-4^ | 0.003 |
| **IWPCV** | **IWPC_MARS** | 0.001 | 1 | 1 |
| **IWPCV** | **IWPC_BART** | 0.125 | 1 | 8.82x10^-4^ |
| **IWPCV** | **NLM** | 2.42x10^-16^ | 0.154 | 1.95x10^-16^ |
| **IWPCV** | **SVR** | 1.31x10^-15^ | 1 | 1.56x10^-15^ |
| **IWPCV** | **MARS** | 6.62x10^-11^ | 1 | 1.5x10^-13^ |
| **IWPCV** | **BART** | 1.82x10^-14^ | 0.958 | 1.76x10^-15^ |
| **IWPC_SVR** | **IWPC_MARS** | 6.62X10^-4^ | 9.22x10^-5^ | 0.055 |
| **IWPC_SVR** | **IWPC_BART** | 0.008 | 0.022 | 2.87X10^-9^ |
| **IWPC_SVR** | **NLM** | 6.16x10^-16^ | 1.32x10^-7^ | 1.72x10^-14^ |
| **IWPC_SVR** | **SVR** | 1.27x10^-15^ | 1.91x10^-5^ | 1.82x10^-13^ |
| **IWPC_SVR** | **MARS** | 1.61x10^-9^ | 0.005 | 2.79x10^-7^ |
| **IWPC_SVR** | **BART** | 3.67x10^-13^ | 1 | 2.56x10^-11^ |
| **IWPC_MARS** | **IWPC_BART** | 1 | 1 | 0.003 |
| **IWPC_MARS** | **NLM** | 2.08x10^-16^ | 1 | 6.26x10^-16^ |
| **IWPC_MARS** | **SVR** | 1.53x10^-16^ | 1 | 1.01x10^-14^ |
| **IWPC_MARS** | **MARS** | 2.71x10^-14^ | 1 | 2.47x10^-13^ |
| **IWPC_MARS** | **BART** | 1.41x10^-15^ | 0.094 | 6.95x10^-15^ |
| **IWPC_BART** | **NLM** | 1.73x10^-16^ | 0.071 | 2.52x10^-16^ |
| **IWPC_BART** | **SVR** | 7.52x10^-16^ | 1 | 1.13x10^-15^ |
| **IWPC_BART** | **MARS** | 6.34x10^-13^ | 1 | 2.46x10^-14^ |
| **IWPC_BART** | **BART** | 2.71x10^-15^ | 1 | 1.13x10^-15^ |
| **NLM** | **SVR** | 0.029 | 1 | 0.057 |
| **Model 1** | **Model 2** | **IWPC^b^** | **ULLA^b^** | **Merged^b^** |
| **NLM** | **MARS** | 1.07x10^-13^ | 0.605 | 3.6x10^-12^ |
| **NLM** | **BART** | 1.05X10^-7^ | 4.97X10^-6^ | 6.01X10^-4^ |
| **SVR** | **MARS** | 4.21X10^-8^ | 1 | 6.19X10^-5^ |
| **SVR** | **BART** | 0.006 | 0.031 | 1 |
| **MARS** | **BART** | 0.048 | 0.264 | 0.016 |
| IWPC indicates International Warfarin Pharmacogenetics Consortium cohort, ULLA, U.S. Latino and Latin American cohort, Merged, ULLA plus IWPC, IWPCV, IWPC variables, IWPC MARS, IWPC variables in a Multivariate Adaptive Regression Splines, IWPC SVR, IWPC variables in a Support Vector Regression, IWPC BART, IWPC variables in a Bayesian Additive Regression Trees  ^a^ *P-*values provided by pairwise Wilcoxon Signed-Rank tests  ^b^ Bonferroni adjustment used to correct for multiple comparisons | | | | |

| **Table S7. Median Percentage Predicted within 20% of Actual and Mean Absolute Error (MAE) in testing data of sensitivity analysis in the Merged cohort (n = 7,030) Multivariate Imputation by Chained Equations ^a^ for missing data at binary variables.** | | |
| --- | --- | --- |
| **Model** | **Within 20% ^b^** | **MAE (95% CI) ^b^** |
| IWPC **^c^** | 46.65 | 8.26 (7.995-8.53) |
| IWPCV **^c^** | 46.72 | 8.24 (7.975-8.51) |
| IWPC SVR **^c^** | 47.72 | 8.1 (7.84-8.37) |
| IWPC MARS **^c^** | 46.68 | 8.25 (7.99-8.52) |
| IWPC BART **^c^** | 47.07 | 8.11 (7.85-8.37) |
| NLM **^d^** | 48.13 | 8.11 (7.84-8.37) |
| SVR **^d^** | 49.03 | 7.91 (7.64-8.17) |
| MARS **^d^** | 47.61 | 8.16 (7.885-8.42) |
| BART **^d^** | 48.36 | 7.93 (7.67-8.19) |
| CI indicates Confidence Interval, IWPC, International Warfarin Pharmacogenetics Consortium, IWPCV, IWPC variables, IWPC MARS, IWPC variables in a Multivariate Adaptive Regression Splines, IWPC SVR, IWPC variables in a Support Vector Regression, IWPC BART, IWPC variables in a Bayesian Additive Regression Trees, NLM, Novel Linear Model  ^a^ **Imputation implemented with the mice package (version 3.13.0) in R using default parameters**  ^b^ Estimates of mean absolute error (MAE) and the percentage of individuals predicted within 20% of their actual dose for each model were based on 100 replicates of resampling testing data.  **^c^** Models feature the clinical variables age, height, weight, race, amiodarone use, and enzyme inducer use and genetic variables *CYP2C9* diplotype, *VKORC1* genotype  **^d^** Models feature the same variables as b in addition to warfarin indication, ethnicity, statin use, aspirin use, history of diabetes | | |

| **Table S8. Median Percentage Predicted within 20% of Actual and Mean Absolute Error (MAE) in sensitivity analysis of complete-case cohort (n = 3,420).** | | |
| --- | --- | --- |
| **Model** | **Within 20%^a^** | **MAE (95% CI)^a^** |
| IWPC **^b^** | 45.13 | 9.15 (8.58-9.71) |
| IWPCV **^b^** | 47.47 | 8.70 (8.17-9.22) |
| IWPC SVR **^b^** | 47.61 | 8.65 (8.12-9.18) |
| IWPC MARS **^b^** | 46.93 | 8.71 (8.20-9.24) |
| IWPC BART **^b^** | 47.27 | 8.69 (8.18-9.21) |
| CI indicates Confidence Interval, IWPC, International Warfarin Pharmacogenetics Consortium, IWPCV, IWPC variables, IWPC MARS, IWPC variables in a Multivariate Adaptive Regression Splines, IWPC SVR, IWPC variables in a Support Vector Regression, IWPC BART, IWPC variables in a Bayesian Additive Regression Trees  **^a^** Estimates of mean absolute error (MAE) and the percentage of individuals predicted within 20% of their actual dose for each model were based on 100 replicates of resampling testing data.  **^b^** Models feature the clinical variables age, height, weight, race, amiodarone use, and enzyme inducer use and genetic variables *CYP2C9* diplotype, *VKORC1* genotype | | |

| **Table S9. Model comparisons by actual-dose groups in the ULLA cohort (n = 1,734).** | | | | | | | |
| --- | --- | --- | --- | --- | --- | --- | --- |
|  | **High (n = 189)** | | **Intermediate (n = 1,128)** | | **Low (n = 417)** | | |
| **Model** | **Within 20%^a^** | **MAE (95% CI) ^a^** | **Within 20% ^a^** | **MAE (95% CI)^a^** | **Within 20% ^a^** | **MAE (95% CI)^a^** | |
| IWPC **^b^** | 24.44 | 19.69 (15.76-23.63) | 59.26 | 6.44 (5.89-6.98) | 27.91 | 7.59 (6.58-8.59) | |
| IWPCV **^b^** | 22.63 | 20.01 (16.02-24.01) | 60.66 | 6.19 (5.66-6.71) | 21.94 | 8.32 (7.3-9.35) | |
| IWPC SVR **^b^** | 21.76 | 20.48 (16.41-24.55) | 60.29 | 6.17 (5.64-6.69) | 20.87 | 8.32 (7.36-9.28) | |
| IWPC MARS **^b^** | 24.81 | 19.87 (15.82-23.91) | 60.62 | 6.19 (5.66-6.72) | 21.84 | 8.32 (7.3-9.34) | |
| IWPC BART **^b^** | 27.27 | 19.4 (15.27-23.53) | 60.02 | 6.23 (5.69-6.76) | 21.07 | 8.35 (7.36-9.34) | |
| NLM **^c^** | 25.66 | 19.52 (15.5-23.54) | 60.18 | 6.18 (5.65-6.71) | 23.42 | 8.22 (7.18-9.26) | |
| SVR **^c^** | 21.62 | 20.54 (16.51-24.57) | 61.3 | 6.13 (5.62-6.65) | 21.21 | 8.3 (7.33-9.26) | |
| MARS **^c^** | 24.28 | 19.95 (15.9-24.01) | 60.52 | 6.17 (5.65-6.7) | 21.77 | 8.33 (7.3-9.36) | |
| BART **^c^** | 26.69 | 19.38 (15.27-23.48) | 59.4 | 6.24 (5.71-6.77) | 21.85 | 8.35 (7.32-9.37) | |
| ULLA indicates U.S. Latino and Latin American warfarin users cohort; CI, Confidence Interval; IWPC, International Warfarin Pharmacogenetics Consortium model; IWPCV, IWPC variables; IWPC MARS, IWPC variables in a Multivariate Adaptive Regression Splines; IWPC SVR, IWPC variables in a Support Vector Regression; IWPC BART, IWPC variables in a Bayesian Additive Regression Trees; NLM, Novel Linear Model  **^a^** Estimates of mean absolute error (MAE) and the percentage of individuals predicted within 20% of their actual dose for each model were based on 100 replicates of resampling 30% testing data.  **^b^** Models feature the variables age, height, weight, race, amiodarone use, and enzyme inducer use and genetic variables *CYP2C9* diplotype, *VKORC1* genotype  **^c^** Models feature the same variables as b in addition to warfarin indication, ethnicity, statin use, aspirin use, history of diabetes  **^d^** Model features the clinical variables only from b | | | | | | | |

| **Table S10. Pairwise Bonferroni Adjusted *P*-values of Median Percentage Predicted Within 20% of Actual Dose in the ULLA Cohort by actual-dose groups ^a^** | | | | |
| --- | --- | --- | --- | --- |
| **Model 1** | **Model 2** | **High^b^** | **Intermediate^b^** | **Low^b^** |
| **IWPC** | **IWPCV** | 1.23x10^-6^ | 7.92x10^-11^ | 1.42x10^-16^ |
| **IWPC** | **IWPC_SVR** | 9.36x10^-8^ | 1.05x10^-5^ | 1.93x10^-16^ |
| **IWPC** | **IWPC_MARS** | 1 | 9.4x10^-9^ | 2.52x10^-16^ |
| **IWPC** | **IWPC_BART** | 3.11x10^-8^ | 0.007 | 1.56x10^-16^ |
| **IWPC** | **NLM** | 0.45 | 8.24x10^-04^ | 1.23x10^-15^ |
| **IWPC** | **SVR** | 6.08x10^-5^ | 4.61x10^-11^ | 3.78x10^-16^ |
| **IWPC** | **MARS** | 1 | 8.5x10^-07^ | 3.04x10^-16^ |
| **IWPC** | **BART** | 4.21x10^-4^ | 1 | 4.39x10^-16^ |
| **IWPCV** | **IWPC_SVR** | 0.221 | 1 | 0.038 |
| **IWPCV** | **IWPC_MARS** | 1.18x10^-6^ | 1 | 1 |
| **IWPCV** | **IWPC_BART** | 7.96x10^-14^ | 0.009 | 0.111 |
| **IWPCV** | **NLM** | 1.31x10^-7^ | 0.159 | 8.14x10^-6^ |
| **IWPCV** | **SVR** | 1 | 0.105 | 1 |
| **IWPCV** | **MARS** | 9.86x10^-4^ | 1 | 1 |
| **IWPCV** | **BART** | 2.59x10^-11^ | 3.03x10^-6^ | 1 |
| **IWPC_SVR** | **IWPC_MARS** | 4.82x10^-9^ | 1 | 0.01 |
| **IWPC_SVR** | **IWPC_BART** | 2.34x10^-14^ | 1 | 1 |
| **IWPC_SVR** | **NLM** | 1.45x10^-9^ | 1 | 5.04x10^-10^ |
| **IWPC_SVR** | **SVR** | 1 | 7.6x10^-6^ | 1 |
| **IWPC_SVR** | **MARS** | 1.85x10^-6^ | 1 | 0.213 |
| **IWPC_SVR** | **BART** | 1.74x10^-12^ | 0.002 | 0.076 |
| **IWPC_MARS** | **IWPC_BART** | 9.11x10^-6^ | 0.17 | 0.12 |
| **IWPC_MARS** | **NLM** | 1 | 0.702 | 4.32x10^-7^ |
| **IWPC_MARS** | **SVR** | 2.44x10^-7^ | 0.181 | 1 |
| **IWPC_MARS** | **MARS** | 1 | 1 | 1 |
| **IWPC_MARS** | **BART** | 0.006 | 5.15x10^-6^ | 1 |
| **IWPC_BART** | **NLM** | 0.143 | 1 | 5.62x10^-10^ |
| **IWPC_BART** | **SVR** | 4.39x10^-13^ | 1.83x10^-6^ | 1 |
| **IWPC_BART** | **MARS** | 1.03x10^-6^ | 0.803 | 0.67 |
| **IWPC_BART** | **BART** | 1 | 0.163 | 0.107 |
| **NLM** | **SVR** | 6.23x10^-11^ | 2.07x10^-7^ | 4.82x10^-10^ |
| **Model 1** | **Model 2** | **High^b^** | **Intermediate^b^** | **Low^b^** |
| **NLM** | **MARS** | 0.216 | 0.907 | 2.35x10^-7^ |
| **NLM** | **BART** | 0.464 | 0.003 | 1.94x10^-7^ |
| **SVR** | **MARS** | 2.95x10^-5^ | 0.006 | 1 |
| **SVR** | **BART** | 4.97x10^-13^ | 2.62x10^-11^ | 0.666 |
| **MARS** | **BART** | 1.01x10^-4^ | 1.03x10^-5^ | 1 |
| IWPC indicates International Warfarin Pharmacogenetics Consortium cohort, ULLA, U.S. Latino and Latin American cohort, Merged, ULLA plus IWPC, IWPCV, IWPC variables, IWPC MARS, IWPC variables in a Multivariate Adaptive Regression Splines, IWPC SVR, IWPC variables in a Support Vector Regression, IWPC BART, IWPC variables in a Bayesian Additive Regression Trees  ^a^ *P-*values provided by pairwise Wilcoxon Signed-Rank tests  ^b^ Bonferroni adjustment used to correct for multiple comparisons | | | | |

**Table S10. Pairwise Bonferroni Adjusted *P*-values of Median Percentage Predicted Within 20% of Actual in the ULLA cohort by race ^a^**

| Model 1 | Model 2 | White ^b^ | Black ^b^ | Mixed/Missing ^b^ |
| --- | --- | --- | --- | --- |
| IWPC | IWPCV | 2.98x10^-11^ | 1 | 1 |
| IWPC | IWPC SVR | 1.3x10^-10^ | 0.086 | 1 |
| IWPC | IWPC MARS | 0.005 | 1 | 1 |
| IWPC | IWPC BART | 0.015 | 1.2x10^-4^ | 1 |
| IWPC | NLM | 8.78x10^-7^ | 1 | 3.64x10^-4^ |
| IWPC | SVR | 2.06x10^-7^ | 1 | 1 |
| IWPC | MARS | 3.19x10^-5^ | 1 | 1 |
| IWPC | BART | 4.08x10^-8^ | 0.008 | 1 |
| IWPC | CLINICAL | 1.78x10^-16^ | 1.78x10^-14^ | 2.48x10^-16^ |
| IWPCV | IWPC SVR | 1 | 0.002 | 0.089 |
| IWPCV | IWPC MARS | 0.002 | 1 | 1 |
| IWPCV | IWPC BART | 0.01 | 2.53x10^-6^ | 1 |
| IWPCV | NLM | 1 | 1 | 0.002 |
| IWPCV | SVR | 1 | 1 | 1 |
| IWPCV | MARS | 0.405 | 1 | 0.41 |
| IWPCV | BART | 1 | 0.01 | 1 |
| IWPCV | CLINICAL | 1.78x10-16 | 1.96x10^-14^ | 3.81x10^-16^ |
| IWPC SVR | IWPC MARS | 0.001 | 1 | 1 |
| IWPC SVR | IWPC BART | 0.001 | 1 | 1 |
| IWPC SVR | NLM | 1 | 2.7x10^-4^ | 5.58x10^-6^ |
| IWPC SVR | SVR | 1 | 1.14x10^-4^ | 0.002 |
| IWPC SVR | MARS | 0.014 | 0.508 | 1 |
| IWPC SVR | BART | 1 | 1 | 0.164 |
| IWPC SVR | CLINICAL | 1.78x10-16 | 1.07x10^-11^ | 5.94x10^-16^ |
| IWPC MARS | IWPC BART | 1 | 0.002 | 1 |
| IWPC MARS | NLM | 1 | 0.513 | 1.08x10^-4^ |
| IWPC MARS | SVR | 0.203 | 1 | 0.148 |
| IWPC MARS | MARS | 1 | 1 | 1 |
| IWPC MARS | BART | 0.099 | 0.558 | 1 |
| IWPC MARS | CLINICAL | 1.78x10-16 | 1.49x10^-13^ | 5.94x10^-16^ |
| IWPC BART | NLM | 1 | 1.28x10^-6^ | 2.3x10^-4^ |
| IWPC BART | SVR | 0.322 | 1.23x10^-5^ | 0.221 |
| Model 1 | Model 2 | white ^b^ | Black ^b^ | Mixed/Missing ^b^ |
| IWPC BART | MARS | 1 | 0.002 | 1 |
| IWPC BART | BART | 0.089 | 1 | 1 |
| IWPC BART | CLINICAL | 1.78x10^-16^ | 2.41x10-11 | 2.72x10^-15^ |
| NLM | SVR | 1 | 1 | 0.427 |
| NLM | MARS | 1 | 0.617 | 3.09x10^-5^ |
| NLM | BART | 1 | 1.65x10^-6^ | 0.002 |
| NLM | CLINICAL | 1.78x10^-16^ | 7.47x10^-15^ | 2.6x10^-16^ |
| SVR | MARS | 1 | 0.27 | 0.055 |
| SVR | BART | 1 | 1.73x10^-4^ | 1 |
| SVR | CLINICAL | 1.78x10^-16^ | 9.54x10^-14^ | 2.77x10^-16^ |
| MARS | BART | 0.76 | 0.403 | 1 |
| MARS | CLINICAL | 1.78x10^-16^ | 1.85x10^-13^ | 4.36x10^-16^ |
| BART | CLINICAL | 1.78x10^-16^ | 1.27x10^-11^ | 1.84x10^-16^ |
| IWPC indicates International Warfarin Pharmacogenetics Consortium cohort, ULLA, U.S. Latino and Latin American cohort, Merged, ULLA plus IWPC, IWPCV, IWPC variables, IWPC MARS, IWPC variables in a Multivariate Adaptive Regression Splines, IWPC SVR, IWPC variables in a Support Vector Regression, IWPC BART, IWPC variables in a Bayesian Additive Regression Trees  ^a^ *P-*values provided by pairwise Wilcoxon Signed-Rank tests  ^b^ Bonferroni adjustment used to correct for multiple comparisons | | | | |

| **Table S11. Model comparisons by country of enrollment data in the ULLA cohort (n = 1,734).** | | | | | | | | | |
| --- | --- | --- | --- | --- | --- | --- | --- | --- | --- |
|  | **Brazil (n =1,190)** | | **Colombia (n =149)** | | **Puerto Rico (n =258)** | | **United States (n = 137)** | |  |
| **Model** | **Within 20%^a^** | **MAE (95% CI) ^a^** | **Within 20% ^a^** | **MAE (95% CI)^a^** | **Within 20% ^a^** | **MAE (95% CI)^a^** | **Within 20% ^a^** | **MAE (95% CI)^a^** |  |
| IWPC **^b^** | 47.13 | 8.18 (7.36-8.99) | 51.59 | 7.25 (5.5-9.01) | 48.97 | 8.15 (6.68-9.63) | 49.73 | 8.87 (5.03-12.71) |  |
| IWPCV **^b^** | 46.30 | 8.21 (7.39-9.03) | 53.05 | 7.05 (5.33-8.77) | 48.16 | 8.26 (6.78-9.74) | 47.66 | 9.26 (5.28-13.24) |  |
| IWPC SVR **^b^** | 45.76 | 8.23 (7.41-9.04) | 52.19 | 7.17 (5.44-8.9) | 46.94 | 8.33 (6.81-9.85) | 48.02 | 9.37 (5.28-13.45) |  |
| IWPC MARS **^b^** | 46.56 | 8.17 (7.36-8.99) | 52.67 | 7 (5.22-8.78) | 48.06 | 8.29 (6.8-9.78) | 47.92 | 9.4 (5.39-13.41) |  |
| IWPC BART **^b^** | 46.50 | 8.13 (7.33-8.94) | 52.51 | 7.17 (5.34-8.99) | 47.13 | 8.27 (6.76-9.78) | 47.17 | 9.36 (5.29-13.44) |  |
| NLM **^c^** | 46.18 | 8.15 (7.35-8.95) | 54.16 | 6.95 (5.22-8.69) | 49.15 | 8.04 (6.5-9.58) | 50.53 | 9.21 (5.19-13.23) |  |
| SVR **^c^** | 46.34 | 8.22 (7.41-9.03) | 53.91 | 7.04 (5.31-8.77) | 46.96 | 8.27 (6.74-9.81) | 50.07 | 9.33 (5.28-13.38) |  |
| MARS **^c^** | 46.48 | 8.18 (7.36-8.99) | 51.99 | 7.06 (5.28-8.84) | 47.93 | 8.24 (6.74-9.75) | 48.01 | 9.41 (5.38-13.43) |  |
| BART **^c^** | 45.72 | 8.17 (7.37-8.97) | 53.61 | 7.11 (5.28-8.94) | 46.86 | 8.17 (6.63-9.72) | 49.31 | 9.39 (5.28-13.5) |  |
| CLINICAL**^d^** | 36.91 | 9.95 (9.02-10.89) | 37.98 | 9.08 (7.03-11.13) | 40.81 | 9.56 (7.93-11.18) | 38.55 | 10.88 (6.83-14.93) |  |
| ULLA indicates U.S. Latino and Latin American warfarin users cohort; CI, Confidence Interval; IWPC, International Warfarin Pharmacogenetics Consortium model; IWPCV, IWPC variables; IWPC MARS, IWPC variables in a Multivariate Adaptive Regression Splines; IWPC SVR, IWPC variables in a Support Vector Regression; IWPC BART, IWPC variables in a Bayesian Additive Regression Trees; NLM, Novel Linear Model  **^a^** Estimates of mean absolute error (MAE) and the percentage of individuals predicted within 20% of their actual dose for each model were based on 100 replicates of resampling 30% testing data.  **^b^** Models feature the variables age, height, weight, race, amiodarone use, and enzyme inducer use and genetic variables *CYP2C9* diplotype, *VKORC1* genotype  **^c^** Models feature the same variables as b in addition to warfarin indication, ethnicity, statin use, aspirin use, history of diabetes  **^d^** Model features the clinical variables only from b | | | | | | | | |  |

**Table S12. Pairwise Bonferroni Adjusted *P*-values of Median Percentage Predicted Within 20% of Actual in the ULLA cohort by country of enrollment ^a^**

| Model 1 | Model 2 | Brazil ^b^ | Colombia ^b^ | Puerto Rico ^b^ | United States ^b^ |
| --- | --- | --- | --- | --- | --- |
| IWPC | IWPCV | 3.92x10^-6^ | 0.004 | 0.585 | 6.26x10^-5^ |
| IWPC | IWPC SVR | 2.25x10^-9^ | 1 | 8.6x10^-6^ | 0.034 |
| IWPC | IWPC MARS | 0.051 | 0.262 | 0.373 | 0.002 |
| IWPC | IWPC BART | 0.054 | 1 | 9.54x10^-5^ | 9.5x10^-5^ |
| IWPC | NLM | 7.42x10^-5^ | 1.67x10^-5^ | 1 | 1 |
| IWPC | SVR | 0.014 | 9.54x10^-4^ | 1.12x10^-4^ | 1 |
| IWPC | MARS | 0.035 | 1 | 0.513 | 0.01 |
| IWPC | BART | 9.27x10^-8^ | 0.003 | 5.13x10^-5^ | 1 |
| IWPC | CLINICAL | 1.78x10^-16^ | 3.66x10^-16^ | 3.52x10^-15^ | 2.01x10^-15^ |
| IWPCV | IWPC SVR | 0.103 | 1 | 0.068 | 1 |
| IWPCV | IWPC MARS | 1 | 1 | 1 | 1 |
| IWPCV | IWPC BART | 1 | 1 | 0.199 | 1 |
| IWPCV | NLM | 1 | 0.922 | 0.796 | 5.31x10^-5^ |
| IWPCV | SVR | 1 | 1 | 0.118 | 0.011 |
| IWPCV | MARS | 1 | 0.234 | 1 | 1 |
| IWPCV | BART | 0.09 | 1 | 0.076 | 0.581 |
| IWPCV | CLINICAL | 1.78x10^-16^ | 4.43x10^-16^ | 3.24x10^-14^ | 4.18x10^-14^ |
| IWPC SVR | IWPC MARS | 3.63x10^-4^ | 1 | 0.083 | 1 |
| IWPC SVR | IWPC BART | 0.003 | 1 | 1 | 1 |
| IWPC SVR | NLM | 1 | 0.029 | 3.97x10^-5^ | 8.42x10^-04^ |
| IWPC SVR | SVR | 0.077 | 0.045 | 1 | 0.069 |
| IWPC SVR | MARS | 0.005 | 1 | 0.228 | 1 |
| IWPC SVR | BART | 1 | 0.3 | 1 | 1 |
| IWPC SVR | CLINICAL | 1.78x10^-16^ | 1.08x10^-15^ | 9.18x10^-13^ | 3.23x10^-13^ |
| IWPC MARS | IWPC BART | 1 | 1 | 0.286 | 1 |
| IWPC MARS | NLM | 1 | 0.144 | 0.374 | 9.76x10^-4^ |
| IWPC MARS | SVR | 1 | 0.886 | 0.377 | 0.048 |
| IWPC MARS | MARS | 1 | 1 | 1 | 1 |
| IWPC MARS | BART | 0.001 | 1 | 0.219 | 1 |
| IWPC MARS | CLINICAL | 1.78x10^-16^ | 6.03x10^-16^ | 1.16x10^-14^ | 7.52x10^-14^ |
| IWPC BART | NLM | 1 | 0.095 | 7.92x10^-4^ | 2.82x10^-6^ |
| Model 1 | Model 2 | Brazil ^b^ | Colombia ^b^ | Puerto Rico ^b^ | United States ^b^ |
| IWPC BART | SVR | 1 | 0.553 | 1 | 2.9x10^-4^ |
| IWPC BART | MARS | 0.133 | 1 | 1 | 1 |
| IWPC BART | BART | 1.78x10^-16^ | 0.886 | 1 | 0.031 |
| IWPC BART | CLINICAL | 1 | 7.02x10^-16^ | 6.88x10^-13^ | 1.22x10^-12^ |
| NLM | SVR | 0.094 | 1 | 2.21x10^-7^ | 1 |
| NLM | MARS | 1.78x10^-16^ | 8.1x10^-4^ | 0.004 | 0.002 |
| NLM | BART | 0.008 | 1 | 1.87x10^-7^ | 0.342 |
| NLM | CLINICAL | 1.78x10^-16^ | 2.1x10^-16^ | 2.27x10^-14^ | 3.17x10^-15^ |
| SVR | MARS | 1.78x10^-16^ | 0.011 | 0.2 | 0.027 |
| SVR | BART | 0.133 | 1 | 1 | 1 |
| SVR | CLINICAL | 1.78x10^-16^ | 2.01x10^-16^ | 8.78x10^-13^ | 7.61x10^-15^ |
| MARS | BART | 1 | 0.016 | 0.154 | 1 |
| MARS | CLINICAL | 0.094 | 6.8x10^-16^ | 2.93x10^-13^ | 2.3x10^-14^ |
| BART | CLINICAL | 1.78x10^-16^ | 2.23x10^-16^ | 3.67x10^-12^ | 2.32x10^-14^ |
| IWPC indicates International Warfarin Pharmacogenetics Consortium cohort, ULLA, U.S. Latino and Latin American cohort, Merged, ULLA plus IWPC, IWPCV, IWPC variables, IWPC MARS, IWPC variables in a Multivariate Adaptive Regression Splines, IWPC SVR, IWPC variables in a Support Vector Regression, IWPC BART, IWPC variables in a Bayesian Additive Regression Trees  ^a^ *P-*values provided by pairwise Wilcoxon Signed-Rank tests  ^b^ Bonferroni adjustment used to correct for multiple comparisons | | | | |  |

| **Figure S1. Comparison of Warfarin Dose Prediction Algorithms in the IWPC cohort.** Proportion of patients predicted within 20% of their actual dose is plotted in the IWPC cohort. The boxplot visualizes five summary statistics (the median, 25% and 75% quartiles and two whiskers at 1.5* Interquartile Range). The points indicate the proportion of patients predicted within 20% at each of the 100 rounds of resampling. Models feature IWPC variables or IWPC variables in addition to new predictors. IWPC indicates International Warfarin Pharmacogenetics Consortium model, IWPCV, IWPC variables, IWPC MARS, IWPC variables in a Multivariate Adaptive Regression Splines, IWPC SVR, IWPC variables in a Support Vector Regression, IWPC BART, IWPC variables in a Bayesian Additive Regression Trees, NLM, Novel Linear Model  **** |
| --- |

**REFERENCES**

Asiimwe, I.G., Zhang, E.J., Osanlou, R., Krause, A., Dillon, C., Suarez-Kurtz, G., Zhang, H., Perini, J.A., Renta, J.Y., Duconge, J., Cavallari, L.H., Marcatto, L.R., Beasly, M.T., Perera, M.A., Limdi, N.A., Santos, P.C.J.L., Kimmel, S.E., Lubitz, S.A., Scott, S.A., Kawai, V.K., Jorgensen, A.L., Pirmohamed, M., 2020. Genetic Factors Influencing Warfarin Dose in Black-African Patients: A Systematic Review and Meta-Analysis. Clin. Pharmacol. Ther. 107, 1420–1433. https://doi.org/10.1002/cpt.1755

Awad, M., Khanna, R., 2015. Support Vector Regression, in: Awad, M., Khanna, R. (Eds.), Efficient Learning Machines: Theories, Concepts, and Applications for Engineers and System Designers. Apress, Berkeley, CA, pp. 67–80. https://doi.org/10.1007/978-1-4302-5990-9_4

Chipman, H.A., George, E.I., McCulloch, R.E., 2010. BART: Bayesian additive regression trees. Ann. Appl. Stat. 4, 266–298. https://doi.org/10.1214/09-AOAS285

Friedman, J.H., 1991. Multivariate adaptive regression splines. Ann. Stat. 1–67.

International Warfarin Pharmacogenetics Consortium, Klein, T.E., Altman, R.B., Eriksson, N., Gage, B.F., Kimmel, S.E., Lee, M.-T.M., Limdi, N.A., Page, D., Roden, D.M., Wagner, M.J., Caldwell, M.D., Johnson, J.A., 2009. Estimation of the warfarin dose with clinical and pharmacogenetic data. N. Engl. J. Med. 360, 753–764. https://doi.org/10.1056/NEJMoa0809329

Liu, R., Li, X., Zhang, W., Zhou, H.-H., 2015. Comparison of Nine Statistical Model Based Warfarin Pharmacogenetic Dosing Algorithms Using the Racially Diverse International Warfarin Pharmacogenetic Consortium Cohort Database. PLOS ONE 10, e0135784. https://doi.org/10.1371/journal.pone.0135784

Roche-Lima, A., Roman-Santiago, A., Feliu-Maldonado, R., Rodriguez-Maldonado, J., Nieves-Rodriguez, B.G., Carrasquillo-Carrion, K., Ramos, C.M., da Luz Sant’Ana, I., Massey, S.E., Duconge, J., 2020. Machine Learning Algorithm for Predicting Warfarin Dose in Caribbean Hispanics Using Pharmacogenetic Data. Front. Pharmacol. 10. https://doi.org/10.3389/fphar.2019.01550

Wickham, H., Averick, M., Bryan, J., Chang, W., McGowan, L.D., François, R., Grolemund, G., Hayes, A., Henry, L., Hester, J., Kuhn, M., Pedersen, T.L., Miller, E., Bache, S.M., Müller, K., Ooms, J., Robinson, D., Seidel, D.P., Spinu, V., Takahashi, K., Vaughan, D., Wilke, C., Woo, K., Yutani, H., 2019. Welcome to the tidyverse. J. Open Source Softw. 4, 1686. https://doi.org/10.21105/joss.01686
